# Synthetic study toward vibralactone

**DOI:** 10.3762/bjoc.21.182

**Published:** 2025-11-04

**Authors:** Liang Shi, Jiayi Song, Yiqing Li, Jia-Chen Li, Shuqi Li, Li Ren, Zhi-Yun Liu, Hong-Dong Hao

**Affiliations:** 1 Shaanxi Key Laboratory of Natural Products & Chemical Biology, College of Chemistry & Pharmacy, Northwest A&F University, Yangling, Shaanxi 712100, China

**Keywords:** alkylidene carbene, C–H insertion, total synthesis, vibralactone

## Abstract

A synthetic study toward vibralactone, a potent inhibitor of pancreatic lipase, is reported. The synthesis of the challenging all-carbon quaternary center within the cyclopentene ring was achieved through intramolecular alkylidene carbene C–H insertion.

## Introduction

**Figure 1:**
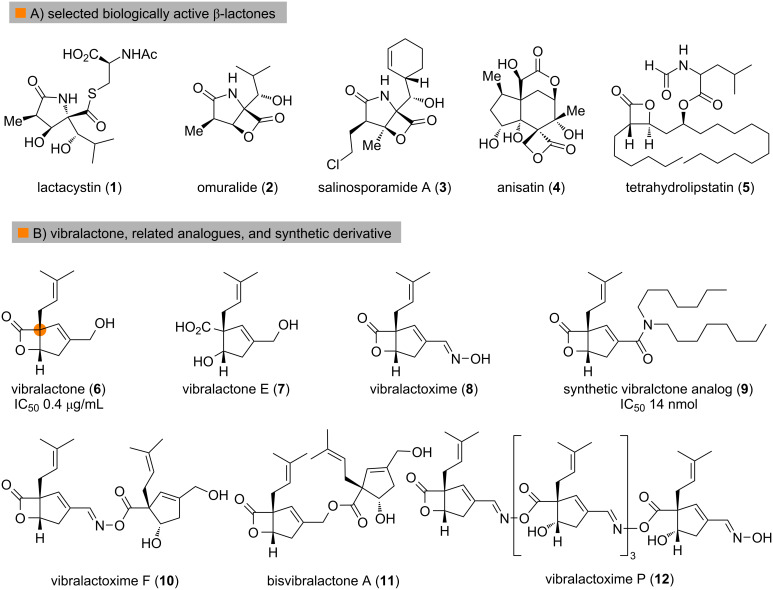
Selected representative natural products and derivatives with β-lactone moiety.

β-Lactones have attracted continuous interest and have been widely utilized as key intermediates in the synthesis of natural products and polymers due to their innate ring strain [1–6]. Moreover, several natural products and their derivatives containing β-lactone as key structural moiety have been isolated and demonstrate potent bioactivities [7] (Figure 1). For example, lactacystin (**1**) which was isolated by Ōmura and co-workers [8–9], is a potent and selective proteasome inhibitor; its active form is the synthetic precursor omuralide (**2**) [10–11]. Similarly, salinosporamide (**3**), a marine natural product isolated by Fenical and co-workers [12], also acts as a proteasome inhibitor and displays more potent in vitro cytotoxicity than omuralide (**2**). Anisatin (**4**), which contains a characteristic spiro β-lactone has been identified as a noncompetitive antagonist of GABA-gated ion channels [13]. Tetrahydrolipstatin (**5**) is a potent pancreatic lipase inhibitor and has been developed into an antiobesity drug marketed under the generic name Orlistat. Vibralactone (**6**), which was isolated by Liu and co-workers from Basidiomycete *Boreostereum vibrans*, features a fused β-lactone with a cyclopentene ring containing an all-carbon quaternary center [14], and inhibits pancreatic lipase with an IC_50_ of 0.4 µg/mL. Several congeners with varying oxidation state, as well as related β-hydroxy acids or esters have also been isolated from the culture broth of the basidiomycete [15–21]. Additionally, a series of vibralactone homodimers and oxime esters **10**–**12** were reported by the groups of Liu and Zhang, respectively [22–23]. Through modification of the primary hydroxy group, a structure-based optimization of vibralactone (**6**) was carried out by Liu and co-workers and yielded several potent pancreatic lipase inhibitors with nanomolar IC_50_ values [24], further supporting vibralactone as a promising lead compound warranting further investigation.

**Scheme 1:**
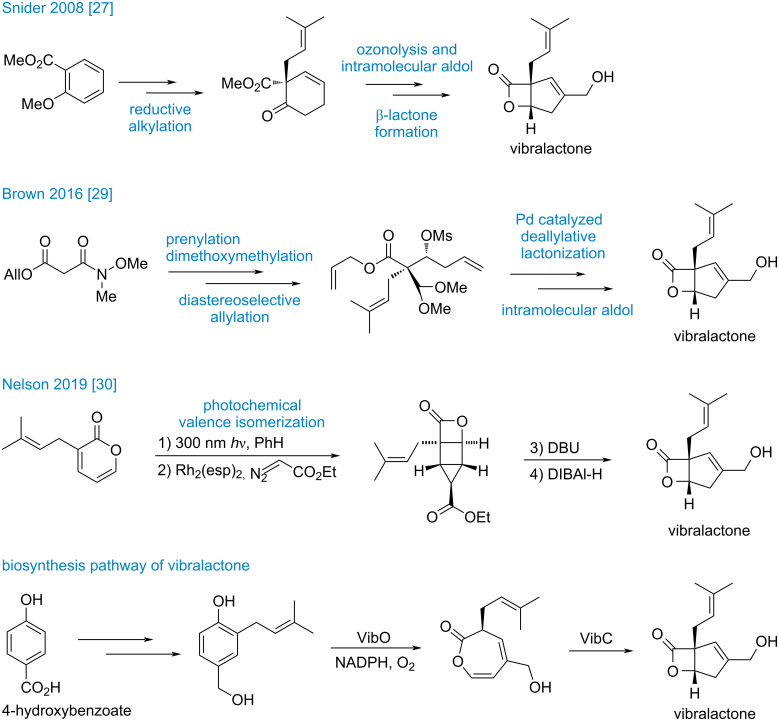
Previous syntheses of vibralactone (**6**).

Although vibralactone (**6**) is a relatively small natural product, its molecular structure features a unique 4/5-fused bicyclic β-lactone with an all-carbon quaternary center and two trisubstituted olefin moieties. It is therefore not surprising that this compound has attracted considerable interest from both the chemical biology and synthetic chemistry communities. Sieber and co-workers disclosed that vibralactone can target ClpP1 and ClpP2 and it could be utilized as a probe to study the activity and structure of the ClpP1P2 complex from *Listeria monocytogenes* [25]. Previously, Snider and co-worker reported the first total synthesis of vibralactone (**6**) employing Birch reductive alkylation, intramolecular aldol reaction and late-stage lactonization as key steps [26] (Scheme 1). Subsequently, they achieved the asymmetric synthesis of vibralactone (**6**) based on the asymmetric Birch reduction–alkylation methodology developed by the Schultz group [27–28]. In 2016, Brown and co-workers described an efficient synthetic route featuring a novel Pd-catalyzed β-lactone formation [29]. In addition to these approaches, Nelson and co-workers reported a very concise and impressive total synthesis of vibralactone involving photochemical valence isomerization of substituted pyrone, cyclopropanation, and ring expansion [30]. Zeng, Liu and co-workers investigated the biosynthetic pathway of vibralactone (**6**). They established that 4-hydroxybenzoate serves as the direct ring precursor of vibralactone and the β-lactone moiety was formed via vibralactone cyclase (VibC)-catalyzed cyclization [31–34]. This is a fascinating cyclization as the all-carbon quaternary center is formed in the last step. Given that the β-lactone moiety may act as a potential covalent inhibitor toward target proteins and that the sterically congested bicyclic skeleton presents a significant synthetic challenge, we herein report our synthetic study toward vibralactone.

**Scheme 2:**
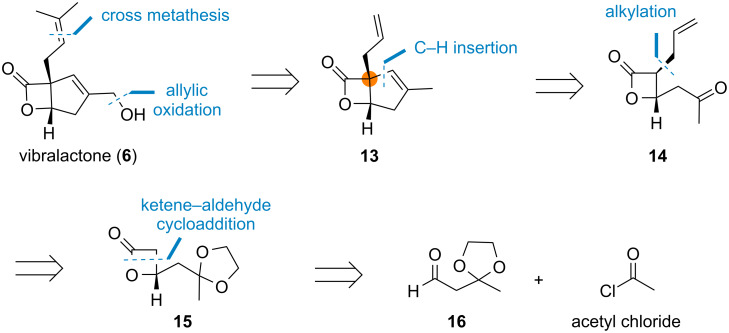
Retrosynthetic analysis of vibralactone (**6**).

Impressed by the unexpected biosynthetic pathway, our synthetic strategy also aimed to construct the quaternary center in the late stage. The retrosynthetic analysis of vibralactone (**6**) is descripted in Scheme 2. Initially, we proposed that vibralactone could be synthesized from lactone **13** through allylic oxidation and cross metathesis. For the construction of the cyclopentene ring, an alkylidene carbene-mediated C–H insertion would be applied [35]. The synthetic route could be traced back to β-lactone **14**, which contains two continuous stereogenic centers with *trans* configuration. This intermediate was intended to be prepared through allylation [36] with its precursor **15** accessible from aldehyde **16** and acetyl chloride through ketene–aldehyde [2 + 2] cycloaddition [37].

## Results and Discussion

**Scheme 3:**
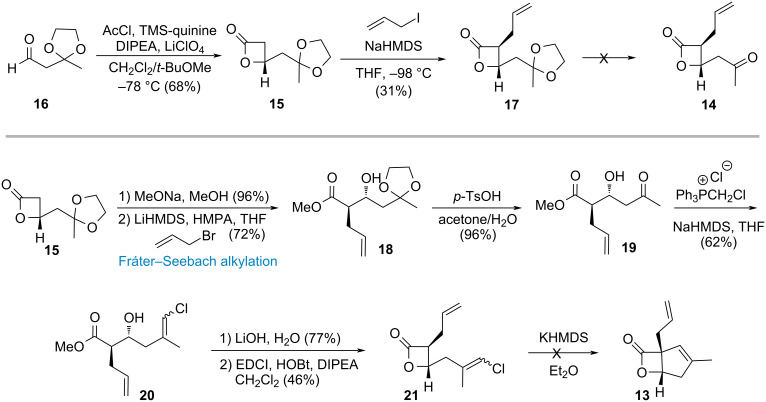
Synthetic study toward vibralactone (**6**) in the present of β-lactone.

Our synthetic route commenced from the known aldehyde **16** which is readily accessed in a single step from commercially available fructone [38] (Scheme 3). Following an efficient *O*-trimethylsilylquinine-catalyzed ketene–aldehyde cycloaddition and subsequent alkylation [36], **17** was synthesized. From **17**, it was envisioned that the bicyclic skeleton could be efficiently constructed through ketal deprotection followed by C–H insertion. However, when attempting to remove the ketal protecting group, only decomposition of the starting material was observed. A plausible explanation for this outcome is that the β-lactone ring, located at the β-position of the methyl ketone, may undergo facile β-elimination, although the corresponding enone product was not isolated. Facing a dead-end, the synthetic route to precursor **14** needed to be revised. From **15**, after sodium methoxide-mediated ring opening of lactone, the Fráter–Seebach alkylation [39–41] was applied to afford β-hydroxy ester **18**. At this stage, the ketal moiety was removed and the resulting intermediate underwent Wittig olefination to yield vinyl chloride **20**. Subsequent hydrolysis and intramolecular esterification furnished intermediate **21**, which was then subjected to C–H insertion [42–44]. To our disappointment, this ring closure still did not proceed to form the all-carbon quaternary center and only decomposition of **21** was observed. The failure is likely due to the sterically hindered environment of the substituted β-lactone ring which precludes the C–H insertion or deprotonation of the β-lactone and interrupted the generation of the alkylidene carbene. Therefore, we modified the synthetic sequence and opted to construct the five-membered ring prior to β-lactone formation, identifying intermediate **19** as a potentially suitable precursor.

**Scheme 4:**
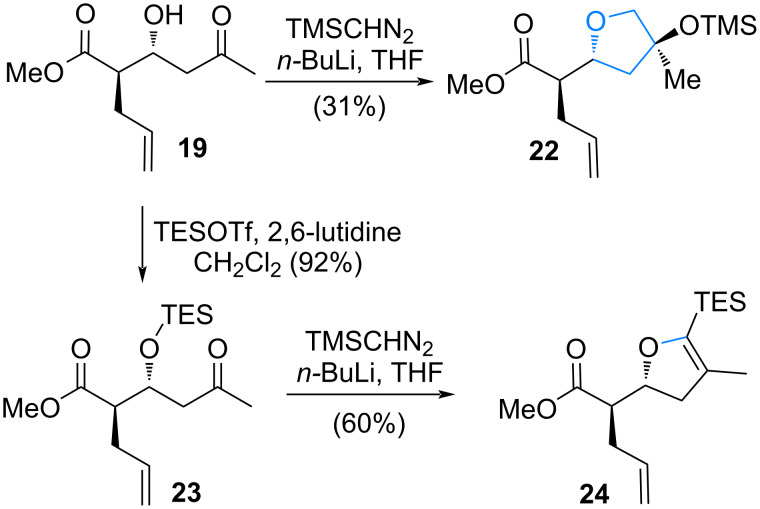
C–H insertion utilizing linear precursor **19**.

From **19**, after treatment with lithiotrimethylsilyldiazomethane [45], only tetrahydrofuran **22** was isolated via a formal [4 + 1] annulation pathway [46] (Scheme 4). Since the hydroxy group interrupted the C–H insertion, it was protected as the TES ether **23** and subjected to the same conditions. However, the reaction only afforded the C–Si insertion product **24** [47].

**Scheme 5:**
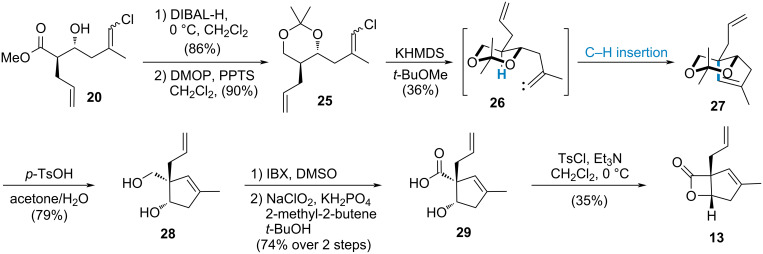
Construction the bicyclic skeleton of vibralactone (**6**) through C–H insertion.

Based on the above results, although the β-lactone was converted into the linear methyl ester **19** to decrease the potential steric hinderance associated with the fused bicyclic skeleton, substrates containing a free hydroxy group or the corresponding TES ether still failed to close the cyclopentene ring. In this scenario, it was necessary to explore additional protecting groups for the hydroxy functionality. Furthermore, given that alkylidene carbenes are electron-deficient and highly electrophilic, electron-rich C–H bonds are more prone to undergo C–H insertion [48]. Following this analysis, commencing from **20**, after reduction, the 1,3-diol intermediate was transformed into acetonide **25** (Scheme 5). Subsequently, the desired five-membered ring was successfully constructed through the in situ-generated alkylidene carbene **26** followed by C–H insertion; herein lies a significant electronic effect influencing this crucial step. With this key intermediate in hand, β-hydroxy acid **29** was synthesized through deprotection, IBX oxidation, and Pinnick–Lindgren–Kraus oxidation and the β-lactone **13** was subsequently obtained through activation of the carboxylic acid. Although we successfully constructed the molecular scaffold of vibralactone (**6**), however, the need to open, reduce, oxidize, and reform the β-lactone lengthened the route beyond initial expectations. Currently an alternative approach towards synthesizing compound **25** is actively being pursued with aims to streamline the overall synthesis.

## Conclusion

In summary, we have developed an approach to assemble the bicyclic skeleton of vibralactone (**6**) utilizing an intramolecular alkylidene carbene C–H insertion as key step. The insights gained from this study illustrate how diverse reactivity patterns and electrophilic characteristics of alkylidene carbenes influence ring closure outcomes. As intermediate **13** may serve as a valuable precursor to vibralactone (**6**) and other congeners such as vibralactone E (**7**), an alternative synthetic route toward **13** is currently being carried out in our laboratory and will be reported in due course.

## Supporting Information

File 1 Characterization data and ^1^H NMR, ^13^C NMR spectra of the compounds.

## Data Availability

All data that supports the findings of this study is available in the published article and/or the supporting information of this article.

## References

[1] Wang X, Wang Z, Ma X, Huang Z, Sun K, Gao X, Fu S, Liu B (2022). Angew Chem, Int Ed.

[2] Guo Z, Bao R, Li Y, Li Y, Zhang J, Tang Y (2021). Angew Chem, Int Ed.

[3] Leverett C A, Purohit V C, Johnson A G, Davis R L, Tantillo D J, Romo D (2012). J Am Chem Soc.

[4] Fukuyama T, Xu L (1993). J Am Chem Soc.

[5] Young M S, LaPointe A M, MacMillan S N, Coates G W (2024). J Am Chem Soc.

[6] Tian J-J, Li R, Quinn E C, Nam J, Chokkapu E R, Zhang Z, Zhou L, Gowda R R, Chen E Y-X (2025). Nature.

[7] Robinson S L, Christenson J K, Wackett L P (2019). Nat Prod Rep.

[8] Ōmura S, Fujimoto T, Otoguro K, Matsuzaki K, Moriguchi R, Tanaka H, Sasaki Y (1991). J Antibiot.

[9] Ōmura S, Matsuzaki K, Fujimoto T, Kosuge K, Furuya T, Fujita S, Nakagawa A (1991). J Antibiot.

[10] Corey E J, Reichard G A, Kania R (1993). Tetrahedron Lett.

[11] Corey E J, Li W-D Z (1999). Chem Pharm Bull.

[12] Feling R H, Buchanan G O, Mincer T J, Kauffman C A, Jensen P R, Fenical W (2003). Angew Chem, Int Ed.

[13] Shenvi R A (2016). Nat Prod Rep.

[14] Liu D-Z, Wang F, Liao T-G, Tang J-G, Steglich W, Zhu H-J, Liu J-K (2006). Org Lett.

[15] Jiang M-Y, Wang F, Yang X-L, Fang L-Z, Dong Z-J, Zhu H-J, Liu J-K (2008). Chem Pharm Bull.

[16] Jiang M-Y, Zhang L, Dong Z-J, Yang Z-L, Leng Y, Liu J-K (2010). Chem Pharm Bull.

[17] Chen H-P, Zhao Z-Z, Yin R-H, Yin X, Feng T, Li Z-H, Wei K, Liu J-K (2014). Nat Prod Bioprospect.

[18] Chen H-P, Jiang M-Y, Zhao Z-Z, Feng T, Li Z-H, Liu J-K (2018). Nat Prod Bioprospect.

[19] Aqueveque P, Céspedes C L, Becerra J, Dávila M, Sterner O (2015). Z Naturforsch, C: J Biosci.

[20] Kang H-S, Kim J-P (2016). J Nat Prod.

[21] Wei J, Li Z-X, Peng G-K, Li X, Chen H-P, Liu J-K (2025). Nat Prod Bioprospect.

[22] Chen H-P, Zhao Z-Z, Li Z-H, Dong Z-J, Wei K, Bai X, Zhang L, Wen C-N, Feng T, Liu J-K (2016). ChemistryOpen.

[23] Liang Y, Li Q, Wei M, Chen C, Sun W, Gu L, Zhu H, Zhang Y (2020). Bioorg Chem.

[24] Wei K, Wang G-Q, Bai X, Niu Y-F, Chen H-P, Wen C-N, Li Z-H, Dong Z-J, Zuo Z-L, Xiong W-Y (2015). Nat Prod Bioprospect.

[25] Zeiler E, Braun N, Böttcher T, Kastenmüller A, Weinkauf S, Sieber S A (2011). Angew Chem, Int Ed.

[26] Zhou Q, Snider B B (2008). Org Lett.

[27] Zhou Q, Snider B B (2008). J Org Chem.

[28] Schultz A G (1999). Chem Commun.

[29] Leeder A J, Heap R J, Brown L J, Franck X, Brown R C D (2016). Org Lett.

[30] Nistanaki S K, Boralsky L A, Pan R D, Nelson H M (2019). Angew Chem, Int Ed.

[31] Zhao P-J, Yang Y-L, Du L, Liu J-K, Zeng Y (2013). Angew Chem, Int Ed.

[32] Yang Y-L, Zhou H, Du G, Feng K-N, Feng T, Fu X-L, Liu J-K, Zeng Y (2016). Angew Chem, Int Ed.

[33] Feng K-N, Yang Y-L, Xu Y-X, Zhang Y, Feng T, Huang S-X, Liu J-K, Zeng Y (2020). Angew Chem, Int Ed.

[34] Feng K-N, Zhang Y, Zhang M, Yang Y-L, Liu J-K, Pan L, Zeng Y (2023). Nat Commun.

[35] Taber D F (2022). Eur J Org Chem.

[36] Parsons P J, Cowell J K (2000). Synlett.

[37] Zhu C, Shen X, Nelson S G (2004). J Am Chem Soc.

[38] Velazquez D G, Luque R (2011). Tetrahedron Lett.

[39] Fráter G (1979). Helv Chim Acta.

[40] Fráter G, Müller U, Günther W (1984). Tetrahedron.

[41] Seebach D, Wasmuth D (1980). Helv Chim Acta.

[42] Grainger R S, Owoare R B (2004). Org Lett.

[43] Esmieu W R, Worden S M, Catterick D, Wilson C, Hayes C J (2008). Org Lett.

[44] Munro K R, Male L, Spencer N, Grainger R S (2013). Org Biomol Chem.

[45] Ohira S, Okai K, Moritani T (1992). J Chem Soc, Chem Commun.

[46] Shen Y, Li L, Pan Z, Wang Y, Li J, Wang K, Wang X, Zhang Y, Hu T, Zhang Y (2015). Org Lett.

[47] Miwa K, Aoyama T, Shioiri T (1994). Synlett.

[48] Grainger R S, Munro K R (2015). Tetrahedron.

