# Supplementary material for: Synthetic study toward vibralactone
Source: Beilstein J Org Chem. 2025 Nov 4;21:2376–82. doi: 10.3762/bjoc.21.182 (PMC12599391; doi:10.3762/bjoc.21.182)
Supplement: File 1 — Characterization data and 1H NMR, 13C NMR spectra of the compounds. [file Beilstein_J_Org_Chem-21-2376-s001.pdf]

BEILSTEIN JOURNAL OF ORGANIC CHEMISTRY

Supporting Information

for

Synthetic study toward vibralactone

Liang Shi, Jiayi Song, Yiqing Li, Jia-Chen Li, Shuqi Li, Li Ren, Zhi-Yun Liu and Hong-Dong Hao

Beilstein J. Org. Chem. **2025,** *21,* 2376–2382. doi:10.3762/bjoc.21.182

Characterization data and ^{1}H NMR, ^{13}C NMR spectra of the compounds

License and Terms: This is a supporting information file under the terms of the Creative Commons Attribution License (https://creativecommons.org/licenses/by/4.0). Please note that the reuse, redistribution and reproduction in particular requires that the author(s) and source are credited and that individual graphics may be subject to special legal provisions.
The license is subject to the *Beilstein Journal of Organic Chemistry* terms and conditions: (https://www.beilstein-journals.org/bjoc/terms)

Table of contents

1. General experimental details S2

1.1. Materials S2

1.2. Instrumentation S3

2. Experimental procedures S4

3. ^{1}H and ^{13}C NMR spectra S15

1. General experimental details

Unless otherwise stated, all reactions were performed with magnetic stirring under a positive pressure of nitrogen or argon gas. Oven-dried glassware (over temperature of 150 °C) was further dried with a heat gun at 650 °C under vacuum, followed by back-filling with inert gas, three times and fitted with rubber septa prior to use. Solids were added under inert gas counter flow or were dissolved and transferred in the appropriate solvent. Solutions and liquid reagents were transferred to reaction vessels by oven-dried stainless-steel cannulas or nitrogen-flushed syringes. Low temperature reactions were carried out in a Dewar vessel filled with acetone/dry ice (−78 °C) or distilled water/ice (0 °C). High-temperature reactions were conducted using a heated silicon oil bath in reaction vessels equipped with a reflux condenser.

1.1 Materials

Dry tetrahydrofuran (THF), dichloromethane (CH_2Cl_2), ethanol (EtOH), toluene (PhMe) and methanol (MeOH) were purchased from Tansoole company and stored over molecular sieves. Ethyl acetate (EtOAc), petroleum ether (PE), methyl *tert*-butyl ether (*t*-BuOMe) used specifically for extraction and flash column chromatography were purchased from commercial sources. All other solvents and reagents were purchased from commercial sources (Sigma Aldrich, Energy chemical, 3A, Adamas).

Reactions were monitored by thin-layer chromatography (TLC) using silica gel F254 pre-coated glass plates (Merck) and visualized by exposure to ultraviolet light (λ = 254 nm) or by staining with aqueous potassium permanganate ($KMnO_4$) solution (7.5 g $KMnO_4$, 50 g K_2CO_3, 6.25 mL aqueous 10% NaOH, 1000 mL distilled H_2O), phosphomolybdic acid hydrate (PMA) solution (10.0 g PMA, 100 mL EtOH) followed by heating with a heat gun (150–600 °C). Flash column chromatography was performed using silica gel (60 Å, 40–63 μm, Merck) and a forced flow of eluent.

1.2 Instrumentation

NMR spectra were recorded on a Bruker Avance III HD 500 MHz and 400 MHz spectrometer equipped with a CryoProbe™. Chemical shifts were reported in parts per million (ppm) respectively to the residual solvent signal (^{1}H NMR: $CDCl_3$: 7.26 ppm; C_6D_6: 7.16 ppm; ^{13}C NMR: $CDCl_3$ 77.2 ppm; C_6D_6: 128.06 ppm). The reported data are represented as follows: chemical shift in parts per million (ppm, δ scale), multiplicity, coupling constants *J* in Hz, integration intensity and proton assignment. Abbreviations used for analysis of multiplets are as follows: s (singlet), br (broad singlet), d (doublet), t (triplet), q (quartet), quin (quintet), h (hextet), and m (multiplet). Variable-temperature NMR spectroscopy was performed at the Northwest A&F University NMR facility.

Mass spectroscopy (MS) experiments were performed in high resolution with an AB SCIEX Triple TOF 5600+ spectrometer (AB SCIEX, Boston, MA, USA). IR spectra were recorded on a Perkin-Elmer Frontier FT-IR spectrometer.

2. Experimental procedures

Synthesis of compound 15

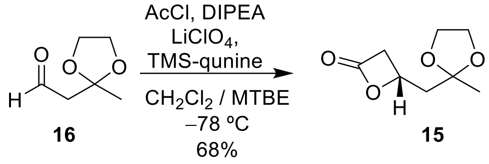

Preparation of TMS-quinine: To a stirred solution of quinine (2.00 g, 6.16 mmol) in dry CH_2Cl_2 (20 mL) at 20 °C was added TMSCl (0.78 mL, 6.16 mmol), the mixture was stirred at 20 °C for 5 h. After the complete transformation of the starting material, evaporation of the solvent and purification of the residue via flash column chromatography (CH_2Cl_2/MeOH 20:1) gave product TMS-quinine (2.19 g, 5.53 mmol, 90%) as a white solid.

To a stirred solution of TMS-quinine (2.33 g, 5.87 mmol) and $LiClO_4$ (3.12 g, 29.33 mmol) in 14 mL of *t*-BuOMe was added 28 mL of CH_2Cl_2 and the reaction mixture was cooled to −78 °C. To the reaction mixture was added *N,N*-diisopropylethylamine (6.06 mL, 36.68 mmol) followed by the aldehyde **16** (1.91 g, 14.67 mmol). Then, a solution of acetyl chloride (2.09 mL, 29.33 mmol) in CH_2Cl_2 (7 mL) was added over 2 hours by a syringe pump. The reaction mixture was stirred for 12 h at −78 °C and quenched by H_2O (20 mL), then extracted with *t*-BuOMe (3 × 20 mL) and the combined organic layers were dried over anhydrous Na_2SO_4 and concentrated in vacuo. The crude product mixture was purified by flash chromatography (petroleum ether/*t*-BuOMe 2:1) to give product **15** (1.72 g, 10.00 mmol, 68%) as a yellow oil.

Data for 15: $\mathbf{R}_f$ = 0.45 (silica, EtOAc : petroleum ether = 1:2, stains with PMA); **^{1}H NMR (400 MHz, Chloroform-*d*):** δ 4.73 – 4.66 (m, 1H), 3.99 – 3.89 (m, 4H), 3.56 (dd, *J* = 16.4, 6.0 Hz, 1H), 3.23 (dd, *J* = 16.4, 4.4 Hz, 1H), 2.37 (dd, *J* = 14.4, 6.0 Hz, 1H), 2.07 (dd, *J* = 14.4, 7.2 Hz, 1H), 1.35 (s, 3H) ppm. **^{13}C NMR (100MHz, Chloroform-*d*):** δ 168.4, 108.2, 67.9, 64.9, 64.8, 44.1, 43.6, 24.6 ppm. **HRMS(ESI)** for $C_8H_{13}O_4^+$ $[M+H]^+$: calcd. 173.0808, found 173.0809. **IR (neat) ν_{max}** 2985, 2890, 1827, 1449, 1213, 1130, 1045, 815, 754 cm^{-1}.

Synthesis of compound S1

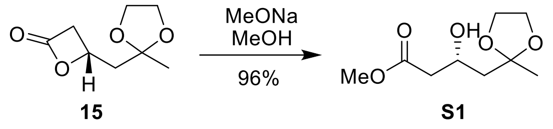

To a stirred solution of **15** (627 mg, 3.64 mmol) in 20 mL MeOH at 32 °C was added MeONa (40 mg, 0.73 mmol). After 30 min, the mixture was concentrated and purified by flash chromatography (EtOAc/petroleum ether 1:2) to afford product **S1** (716 mg, 3.51 mmol, 96%) as a yellow oil.

Data for S1: R*f* = 0.36 (silica, EtOAc : petroleum ether = 1:2, stains with PMA); **^{1}H NMR (400 MHz, Chloroform-*d*):** δ 4.35 – 4.27 (m, 1H), 3.99 – 3.94 (m, 4H), 3.68 (s, 3H), 3.66 (s, 1H), 2.50 (dd, *J* = 15.4, 7.6 Hz, 1H), 2.42 (dd, *J* = 15.6, 5.2 Hz, 1H), 1.86 (d, *J* = 6.0 Hz, 2H), 1.35 (s, 3H) ppm.; **^{13}C NMR (100 MHz, Chloroform-*d*)**: δ 172.2, 110.0, 65.1, 64.7, 64.4, 51.8, 44.5, 41.9, 24.2 ppm; **HRMS ESI** $C_9H_{17}O_5^+$ $[M+H]^+$: calcd. 205.1071, found 205.1075. **IR (neat) ν_{max}** 2985, 2955, 2892, 1737, 1214, 1176, 1141, 1049 cm^{-1}. **Enantiomeric excess:** 48%, determined by HPLC (Daicel Chiralpak OD-H column, hexane/isopropanol = 90/10, flow rate 1 mL/min, 220 nm, 25°C): t_R = 3.992 min (major), t_R = 5.187 min (minor).

Although this cinchona alkaloid-catalyzed acid chloride–aldehyde asymmetric cyclocondensation has been applied in several total syntheses, it unfortunately, in the current case, afforded only 48% ee for β-lactone **15**. So the optical rotation value of all the intermediates reported here are not measured and alternative methods for the synthesis of compound **S1** are still needed.

Synthesis of compound 17

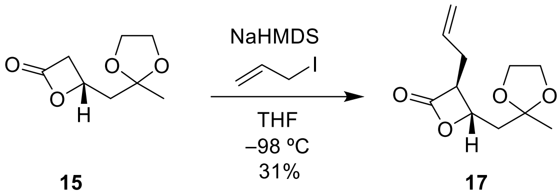

To a stirred solution of the NaHMDS (2.67 mL, 2.67 mmol, 1.0 M solution in THF) in THF (25 mL) at −98 °C was added **15** (438 mg, 2.54 mmol). After stirring for 15 min, allyl iodide (0.35 mL, 3.82 mmol) was added to the reaction mixture and stirred for additional 5 h. The mixture was quenched by saturated NH_4Cl aqueous solution and extracted with EtOAc (3 × 10 mL). The combined organic extract was dried over anhydrous Na_2SO_4 and concentrated in vacuo. The crude product was purified via flash chromatography (EtOAc/petroleum ether 1:5) to provide **17** (165 mg, 0.78 mmol, 31%) as a yellow oil.

Data for 17: **R*f*** = 0.45 (silica, EtOAc : petroleum ether = 1:3, stains with PMA); **^{1}H NMR (500 MHz, Chloroform-*d*):** δ 5.86 – 5.77 (m, 1H), 5.20 – 5.14 (m, 2H), 4.48 – 4.43 (m, 1H), 3.99 – 3.90 (m, 4H), 3.46 – 3.40 (m, 1H), 2.53 (t, *J* = 7.0 Hz, 2H), 2.34 (dd, *J* = 14.5, 6.5 Hz, 1H), 2.08 (dd, *J* = 14.5, 7.0 Hz, 1H), 1.35 (s, 3H) ppm.

Synthesis of compound 18

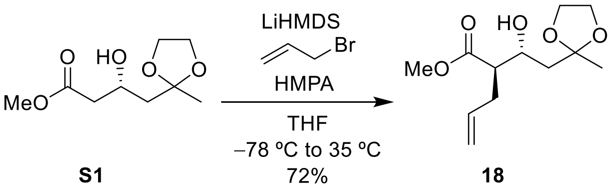

To a stirred solution of **S1** (2.92 g, 4.27 mmol) in dry tetrahydrofuran (80 mL) at −78 °C was added LiHMDS (31.40 mL, 31.40 mmol, 1.0 M solution in THF) dropwise. After 30 min, the mixture was stirred at 35 °C, then allyl bromide (2.09 mL, 22.83 mmol) was added followed by HMPA (6.2 mL, 35.6 mmol). The reaction mixture was stirred at 35 °C for 3 h, then quenched with saturated aqueous NH_4Cl, extracted with *t*-BuOMe (3 × 20 mL), dried over anhydrous Na_2SO_4 and concentrated in vacuo. The residue was purified by flash chromatography (EtOAc/petroleum ether 1:4) to give compound **18** (2.5 g, 10.22 mmol, 72%) as a yellow oil.

Data for 18: **R*f*** = 0.43 (silica, EtOAc : petroleum ether = 1:2, stains with PMA). **^{1}H NMR (400MHz, Chloroform-*d*):** δ 5.78 – 5.66 (m, 1H), 5.06 (dd, *J* = 17.2, 1.6 Hz, 1H), 5.00 (d, *J* = 10.0 Hz, 1H), 4.07 – 4.00 (m, 1H), 3.99 – 3.94 (m, 4H), 3.68 (s, 3H), 3.63 (d, *J* = 2.4 Hz, 1H), 2.56 – 2.49 (m, 1H), 2.42 – 2.25 (m, 2H), 1.94 (dd, *J* = 14.6, 1.6 Hz, 1H), 1.79 (dd, *J* = 14.8, 9.6 Hz, 1H), 1.34 (s, 3H) ppm. **^{13}C NMR (100MHz, Chloroform-*d*):** δ 174.3, 135.2, 117.0, 110.2, 68.8, 64.8, 64.4, 51.6, 51.6, 42.5, 32.8, 24.3 ppm. **HRMS(ESI)** for $C_{12}H_{21}O_5^+$ $[M+H]^+$: calcd. 245.1384, found 245.1382. **IR (neat) ν$_{max}$** 2985, 2953, 2892, 1737, 1441, 1248, 1201, 1170, 1041 cm^{-1}.

Synthesis of compound 19

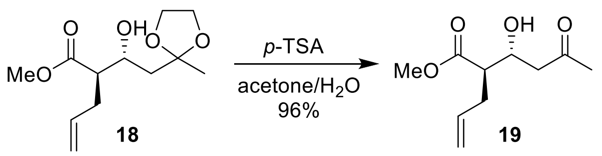

To a stirred solution of compound **18** (1.87 g, 7.66 mmol) in acetone (15 mL) and H_2O (15 mL) were added *p*-TSA (728 mg, 3.83 mmol). The mixture was stirred at 4 °C for 3 h, then quenched with saturated aqueous $NaHCO_3$, extracted with *t*-BuOMe (3 × 20 mL), dried over anhydrous Na_2SO_4 and concentrated in vacuo. The residue was purified by flash chromatography (EtOAc/petroleum ether 1:2) to give compound **19** (1.47 g, 7.35 mmol, 96%) as a yellow oil.

Data for 19: $\mathbf{R}_f$ = 0.43 (silica, EtOAc : petroleum ether = 1:2, stains with PMA). **^{1}H NMR (400MHz, Benzene-*d*$_6$):** δ 5.78 – 5.65 (m, 1H), 5.07 (d, *J* = 17.2 Hz, 1H), 4.97 (d, *J* = 10.4 Hz, 1H), 4.29 – 4.21 (m, 1H), 3.49 – 3.41 (m, 1H), 3.35 (s, 3H), 2.57 – 2.49 (m, 1H), 2.44 – 2.33 (m, 1H), 2.31 – 2.12 (m, 3H), 1.63 (s, 3H) ppm. **^{13}C NMR (100MHz, Benzene-*d*$_6$):** δ 207.3, 173.9, 135.6, 117.1, 68.4, 51.2, 50.9, 47.6, 33.1, 30.2 ppm. **HRMS(ESI)** for $C_{10}H_{17}O_4^+$ $[M+H]^+$: calcd. 201.1121, found 201.1124. **IR (neat) ν_{max}** 1721, 1437, 1365, 1242, 1199, 1168 cm^{-1}.

Synthesis of compound 20

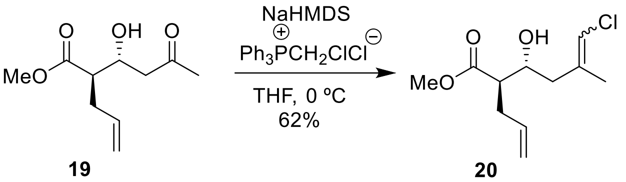

To a stirred solution of (chloromethyl)triphenylphosphonium chloride (1.41 g, 4.06 mmol) in dry tetrahydrofuran (20 mL) at 0 °C was added NaHMDS (2.03 mL, 4.06 mmol, 2.0 M solution in THF), the reaction mixture was stirred for 30 min and a solution of **19** (271 mg, 1.35 mmol) in tetrahydrofuran (5 mL) was added dropwise. After 3 h, the reaction mixture was quenched by saturated aqueous NH_4Cl solution (20 mL) and the aqueous layer was extracted with *t*-BuOMe (3 × 20 mL). The combined organic layers were washed with brine (20 mL) and dried over Na_2SO_4. The solvent was removed by rotary evaporation to give crude product, which was purified through flash column chromatography (EtOAc/petroleum ether 1:30) to yield **20** (199 mg, 0.85 mmol, 62%) as a light-yellow oil. Compound **20** was obtained as inseparable mixture in a ratio of 7:3.

Data for 20: **R$_f$** = 0.75 (silica, EtOAc : petroleum ether = 1:3, stains with PMA). **^{1}H NMR (400MHz, Chloroform-*d*):** δ 5.92 – 5.86 (m, 1H), 5.79 – 5.68 (m, 1H), 5.14 – 5.07 (m, 1H), 5.05 (dd, *J* = 10.2, 1.2 Hz, 1H), 3.96 – 3.90 (m, 0.3H), 3.88 – 3.81 (m, 0.7H), 3.73 – 3.70 (m, 3H), 2.81 (d, *J* = 8.8 Hz, 0.3H), 2.67 (d, *J* = 7.6 Hz, 0.7H), 2.58 – 2.50 (m, 1H), 2.46 – 2.40 (m, 2H), 2.27 – 2.21 (m, 1H), 1.80 (d, *J* = 1.2 Hz, 3H) ppm. **^{13}C NMR (100MHz, Chloroform-*d*):** major isomer: δ 175.0, 135.2, 134.6, 117.7, 114.9, 69.5, 51.9, 49.9, 43.1, 33.8, 16.8 ppm. **HRMS(ESI)** for $C_{11}H_{18}ClO_3^+$ $[M+H]^+$: calcd. 233.0939, found 233.0940. **IR (neat) ν_{max}** 2948, 2921, 1728, 1439, 1168 cm^{-1}.

Synthesis of compound 21

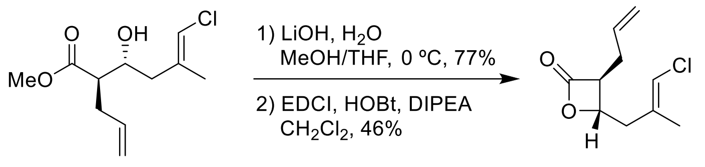

20 **21**

To a stirred solution of **20** (102 mg, 0.44 mmol) in MeOH (1.5 mL) and THF (3 mL) at 0 °C was added LiOH solution (1.10 mL, 4.40 mmol, 4.0 M solution in water), and the reaction mixture was stirred for 3.5 h. Then 1 mol/L HCl was added to the reaction mixture to adjust the pH below 4 and the aqueous layer was extracted with EtOAc (3 × 5 mL). The combined organic layers were washed with brine (10 mL) and dried over Na_2SO_4. The solvent was removed by rotary evaporation to give a crude product, which was purified through flash column chromatography (EtOAc/petroleum ether 1:3) to yield **S3** (73 mg, 0.33 mmol, 77%) as a colorless oil.

To a stirred solution of **S3** (15 mg, 0.07 mmol) in dry CH_2Cl_2 (2 mL) at 36 °C was added EDCI (16 mg, 0.08 mmol), and HOBt (14 mg, 0.10 mmol). After 1 h, DIPEA (14 μL, 0.07 mmol) was added to the reaction. After 4 h, the reaction was quenched with saturated aqueous NH_4Cl solution (5 mL) and the aqueous layer was extracted with *t*-BuOMe (3 × 10 mL). The combined organic layers were washed with brine (10 mL) and dried over Na_2SO_4. The solvent was removed by rotary evaporation to give the crude product, which was purified through flash column chromatography (EtOAc/petroleum ether 1:20) to yield **21** (6.1 mg, 0.03 mmol, 46%) as a yellow oil. Compound **21** was isolated as single diastereoisomer.

Data for 21: R$_f$ = 0.89 (silica, EtOAc : petroleum ether = 1:2, stains with PMA). **^{1}H NMR (400MHz, Chloroform-*d*):** δ 5.98 – 5.93 (m, 1H), 5.80 – 5.68 (m, 1H), 5.20 – 5.16 (m, 1H), 5.16 – 5.13 (m, 1H), 4.39 – 4.32 (m, 1H), 3.36 – 3.30 (m, 1H), 2.67 – 2.46 (m, 4 H), 1.83 (d, *J* = 1.6 Hz, 3H) ppm.

Synthesis of compound 22

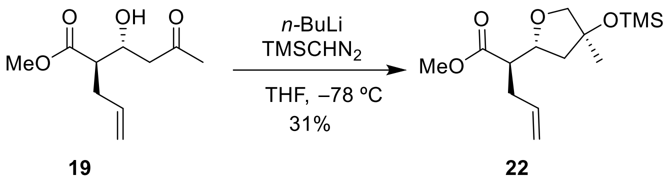

To a stirred solution of $TMSCHN_2$ (0.21 mL, 0.42 mmol) in dry THF (2 mL) at −78 °C was added *n*-BuLi (0.26 mL, 0.42 mmol, 1.6 M solution in hexanes) and the reaction mixture was stirred for 10 min. Then a solution of **19** (47 mg, 0.23 mmol) in THF (1 mL) was added dropwise. After 4 h stirring at −78 °C, the reaction mixture was quenched by 1 mol/L HCl solution (3 mL) and the aqueous layer was extracted with *t*-BuOMe (3 × 5 mL). The combined organic layers were washed with brine (20 mL) and dried over Na_2SO_4. The solvent was removed by rotary evaporation to give crude product, which was purified through flash column chromatography (EtOAc/petroleum ether 1:40) to yield **22** (20.4 mg, 0.07 mmol, 31%) as a light-yellow oil.

Data for 22: $\mathbf{R}_f$ = 0.40 (silica, EtOAc : petroleum ether = 1:40, stains with PMA). **^{1}H NMR (400MHz, Chloroform-*d*):** δ 5.78 – 6.66 (m, 1H), 5.06 (dd, J = 17.2, 1.6 Hz, 1H), 5.00 (d, J = 9.6 Hz, 1H), 4.31 (ddd, J = 10.0, 8.4, 6.0 Hz, 1H), 3.73 (d, J = 9.2 Hz, 1H), 3.69 (s, 3H), 3.63 (d, J = 9.2 Hz, 1H), 2.55 (ddd, J = 10.4, 8.4, 4.4 Hz, 1H), 2.38 – 2.28 (m, 1H), 2.22 – 2.14 (m, 1H), 2.10 (dd, J = 12.6, 6.8 Hz, 1H), 1.53 (dd, J = 12.4, 10.0 Hz, 1H), 1.40 (s, 3H), 0.12 (s, 9H) ppm. **^{13}C NMR (100MHz, Chloroform-*d*):** δ 174.1, 134.9, 117.1, 80.9, 80.4, 79.9, 51.8, 51.5, 46.3, 33.3, 26.1, 2.3 ppm.

The relative stereochemistry of compound **22** was determined through 2D NMR. From the NOESY, the nucleophilic attack from *Re* face was directed by the hydroxy group.

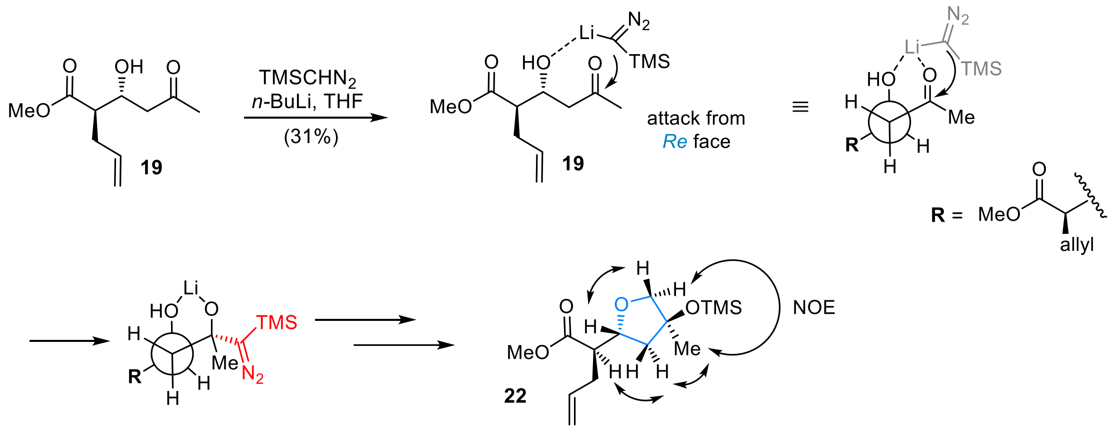

Synthesis of compound 23

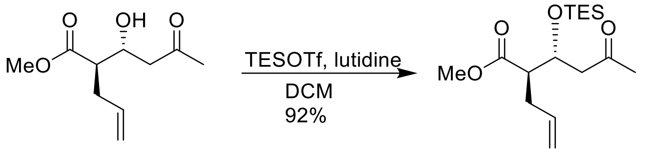

19 23

To a stirred solution of **19** (36 mg, 0.18 mmol) in CH_2Cl_2 (2 mL) at 0 °C was added 2,6-lutidine (32 μL, 0.27 mmol) and TESOTf (53 μL, 0.23 mmol), and the reaction mixture was stirred for 2 h. Then, the reaction mixture was quenched by saturated aqueous NH_4Cl solution (5 mL) and the aqueous layer was extracted with EtOAc (3 × 5 mL). The combined organic layers were washed with brine (10 mL) and dried over Na_2SO_4. The solvent was removed by rotary evaporation to give the crude product, which was purified through flash column chromatography (EtOAc/petroleum ether 1:30) to yield **23** (49 mg, 0.17 mmol, 92%) as a light-yellow oil.

Data for 23: R*f* = 0.81 (silica, EtOAc : petroleum ether = 1:4, stains with PMA). **^{1}H NMR (400MHz, Chloroform-*d*):** δ 5.78 – 5.67 (m, 1H), 5.05 (dd, *J* = 17.2, 1.6 Hz, 1H), 4.99 (d, *J* = 10.4 Hz, 1H), 4.47 (q, *J* = 5.6 Hz, 1H), 3.65 (s, 3H), 2.65 – 2.59 (m, 3H), 2.42 – 2.27 (m, 2H), 2.15 (s, 3H), 0.92 (t, *J* = 8.0 Hz, 9H), 0.58 (q, *J* = 7.6 Hz, 6H) ppm. **^{13}C NMR (100MHz, Chloroform-*d*):** δ 206.8, 173.2, 135.7, 116.5, 68.6, 51.8, 51.5, 48.0, 31.4, 30.9, 6.8, 4.8 ppm.

Synthesis of compound 24

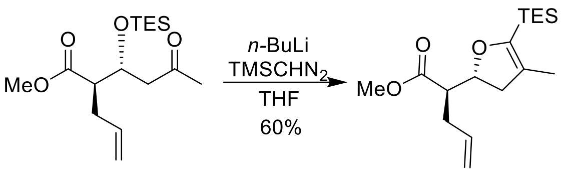

23 24

To a stirred solution of $TMSCHN_2$ (80 μL, 0.16 mmol) in dry THF (1.5 mL) at −78 °C was added *n*-BuLi (0.10 mL, 0.16 mmol, 1.6 M solution in hexanes) and the reaction mixture was stirred for 10 min. Then, a solution of **23** (28 mg, 0.09 mmol) in THF (1 mL) was added dropwise. After 4 h at −78 °C, the reaction mixture was quenched by 1 mol/L HCl solution (2 mL) and the aqueous layer was extracted with *t*-BuOMe (3 × 5 mL). The combined organic layers were washed with brine (20 mL) and dried over Na_2SO_4. The solvent was removed by rotary evaporation to the give crude product, which was purified through flash column chromatography (EtOAc/petroleum ether 1:80) to yield **24** (17 mg, 0.05 mmol, 60%) as a light-yellow oil.

Data for 24: **R*f*** = 0.71 (silica, EtOAc : petroleum ether = 1:20, stains with PMA). **¹H NMR (400MHz, Chloroform-*d*):** δ 5.80 – 5.68 (m, 1H), 5.06 (dd, *J* = 17.2, 1.6 Hz, 1H), 5.00 (d, *J* = 10.0 Hz, 1H), 4.56 (ddd, *J* = 10.0, 8.4, 6.8 Hz, 1H), 3.65 (s, 3H), 2.72 – 2.58 (m, 2H), 2.39 – 2.29 (m, 2H), 2.25 – 2.16 (m, 1H), 1.69 (s, 3H), 0.94 (t, *J* = 8.0 Hz, 9H), 0.64 (q, *J* = 8.0 Hz, 6H) ppm. **¹³C NMR (100MHz, Chloroform-*d*):** δ 173.8, 150.8, 135.1, 120.7, 116.7, 80.7, 51.4, 51.1, 40.5, 32.4, 12.2, 7.3, 3.1 ppm.

Synthesis of compound 25

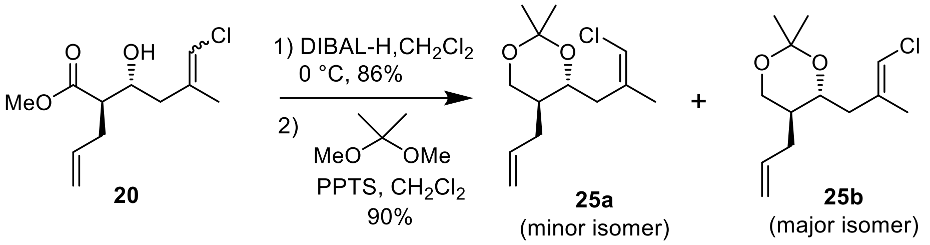

To a stirred solution of alcohol **20** (1.06 g, 4.56 mmol) in dry CH_2Cl_2 (40 mL) at 0 °C was added DIBAL-H (9.13 mL, 9.13 mmol, 1.0 M solution in hexane) under argon. After 4 h, the mixture was quenched by MeOH (3 mL) and then saturated Rochelle's salt solution (30 mL) was added, and the reaction mixture was stirred vigorously for 2 h at room temperature until the two layers were clear. The aqueous phase was extracted with *t*-BuOMe (3 × 30 mL), the combined organic layers were washed with brine, dried over anhydrous Na_2SO_4 and concentrated under reduced pressure. Afterwards, the residue was purified through flash column chromatography (EtOAc/petroleum ether 1:6 to 1:3 to 1:2) to yield **S2** (793 mg, 3.93 mmol, 86%) as a colorless oil.

To a stirred solution of **S2** (793 mg, 3.93 mmol) in CH_2Cl_2 (40 mL) at 13 °C was added 2,2-dimethoxypropane (1.5 mL, 11.80 mmol) and pyridinium *p*-toluenesulfonate (494 mg, 1.97 mmol) and the reaction mixture was stirred for 4 h. Afterwards the mixture was directly concentrated for column chromatography separation (eluent EtOAc/petroleum ether 1:50) to yield **25** (870.7 mg, 3.56 mmol, 90%) as a colorless oil. An analytically pure sample of **25a** and **25b** was obtained from preparative TLC (EtOAc/petroleum ether 1:60).

Data for 25a: Minor isomer R*f* = 0.28 (silica, EtOAc : petroleum ether = 1:30, stains with PMA). **¹H NMR (400MHz, Chloroform-*d*):** δ 5.83 (s, 1H), 5.78 – 5.67 (m, 1H), 5.09 – 5.00 (m, 2H), 3.81 – 3.72 (m, 2H), 3.56 (t, *J* = 11.6 Hz, 1H), 2.65 (dd, *J* = 14.0, 2.8 Hz, 1H), 2.28 – 2.20 (m, 2H), 1.91 – 1.84 (m, 1H), 1.82 (s, 3H), 1.73 – 1.63 (m, 1H), 1.38 (s, 3H), 1.35 (s, 3H) ppm. **¹³C NMR (100MHz, Chloroform-*d*):** δ 137.3, 135.2, 117.1, 112.6, 98.1, 72.6, 63.9, 38.7, 35.8, 33.1, 29.4,

22.6, 19.5 ppm. **IR (neat)** ν_{max} 2990, 2922, 2858, 1378, 1199, 1168, 1124, 1055, 915 cm^{-1}.

Data for 25b: Major isomer R*f* = 0.23 (silica, EtOAc : petroleum ether = 1:30, stains with PMA). **^{1}H NMR (400MHz, Chloroform-*d*):** δ 5.84 (s, 1H), 5.74 – 5.63 (m, 1H), 5.07 – 5.04 (m, 1H), 5.02 (s, 1H), 3.77 (dd, *J* = 12.0, 5.2 Hz, 1H), 3.71 – 3.64 (m, 1H), 3.55 (dd, *J* = 11.8, 10.8 Hz, 1H), 2.38 (d, *J* = 14.0 Hz, 1H), 2.19 – 2.10 (m, 2H), 1.88 – 1.81 (m, 1H), 1.80 (s, 3H), 1.71 – 1.60 (m, 1H), 1.39 (s, 3H), 1.35 (s, 3H) ppm. **^{13}C NMR (100MHz, Chloroform-*d*):** δ 135.9, 134.9, 117.2, 113.7, 98.3, 71.7, 63.8, 40.6, 38.3, 33.1, 29.4, 19.4, 17.2 ppm. **HRMS(ESI)** for $C_{13}H_{22}ClO_2^+$ [M+H]$^+$: calcd. 245.1303, found 245.1302. **IR (neat)** ν_{max} 2990, 2921, 2854, 1376, 1263, 1200, 1165, 1128 cm^{-1}.

The olefin configuration of **25b** was determined through NOESY.

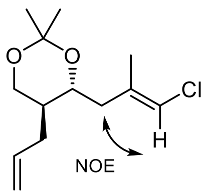

25b

Synthesis of compound 27

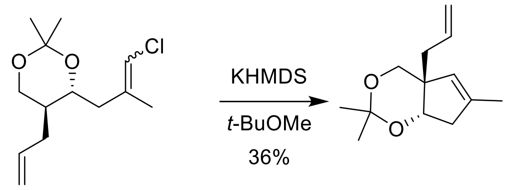

25 **27**

To a stirred solution of **25** (100 mg, 0.41 mmol) in *t*-BuOMe (4 mL) at 4 °C was added KHMDS (2.05 mL, 2.05 mmol, 1.0 M solution in THF) at a rate of 0.034 mm/min by a syringe pump. The reaction mixture was stirred for 3 h, then quenched by saturated aqueous NH_4Cl solution (5 mL), and the aqueous layer was extracted with *t*-BuOMe (3 × 5 mL). The combined organic layers were washed with brine (10 mL) and dried over Na_2SO_4. The solvent was removed by rotary evaporation to give the crude product, which was purified through flash column chromatography (EtOAc/petroleum ether 1:100 to 1:80) to yield **27** (30 mg, 0.15 mmol, 36%) as a colorless oil.

Data for 27: R*f* = 0.49 (silica, EtOAc : petroleum ether = 1:30, stains with PMA). **^{1}H NMR (400MHz, Chloroform-*d*):** δ 5.77 – 5.66 (m, 1H), 5.12 – 5.00 (m, 3H), 4.03 (d, *J* = 6.4 Hz, 1H), 3.56 – 3.49 (m, 2H), 2.68 – 2.59 (m, 1H), 2.26 – 2.12 (m, 3H), 1.76 (s, 3H), 1.39 (s, 3H), 1.32 (s, 3H) ppm. **^{13}C NMR (100MHz, Chloroform-*d*):** δ 140.2, 134.6, 126.7, 117.5, 98.8, 76.1, 64.6, 54.3, 43.6, 40.7, 26.1, 22.8, 16.6 ppm. **HRMS(ESI)** for $C_{10}H_{17}O_2^+$ [M+H]$^+$: calcd. 209.1536, found 209.1537. **IR (neat)** ν_{max} 2925, 2860,1375, 1226, 1195, 1081 cm^{-1}.

Synthesis of compound 28

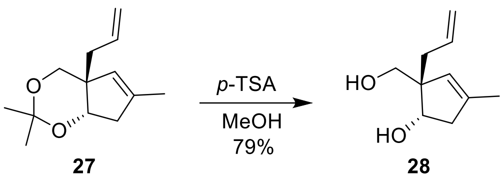

To a stirred solution of **27** (38 mg, 0.18 mmol) in MeOH (2 mL) at 13 °C was added *p*-TSA (34.7 mg, 0.18 mmol) and the reaction mixture was stirred for 5 h. The reaction mixture was quenched by saturated aqueous $NaHCO_3$ solution (5 mL) and the aqueous layer was extracted with EtOAc (3 × 5 mL). The combined organic layers were washed with brine (10 mL) and dried over Na_2SO_4. The solvent was removed by rotary evaporation to give crude product, which was purified through flash column chromatography (EtOAc/petroleum ether 1:4 to 1:2 to 1:1) to yield **28** (24 mg, 0.14 mmol, 79%) as a colorless oil.

Data for 28: R*f* = 0.23 (silica, EtOAc : petroleum ether = 1:2, stains with PMA). **^{1}H NMR (400MHz, Chloroform-*d*):** δ 5.83 – 5.71 (m, 1H), 5.11 – 5.01 (m, 3H), 4.27 (dd, *J* = 7.8, 5.6 Hz, 1H), 3.67 (d, *J* = 11.2 Hz, 1H), 3.62 (d, *J* = 11.2 Hz, 1H), 2.63 (dd, *J* = 16.8, 7.6 Hz, 1H), 2.29 (s, 2H), 2.27 – 2.20 (m, 2H), 2.12 (dd, *J* = 13.8, 8.0 Hz, 1H), 1.73 (s, 3H) ppm. **^{13}C NMR (100MHz, Chloroform-*d*):** δ 140.6, 134.9, 127.0, 117.7, 78.9, 65.9, 56.1, 46.3, 40.2, 17.2 ppm. **HRMS(ESI)** for $C_{13}H_{21}O_2^+$ $[M+H]^+$: calcd. 169.1223, found 169.1224. **IR (neat) ν_{max}** 3382, 2968, 2925, 1713, 1440, 1071, 1034, 1001, 917 cm^{-1}.

Synthesis of compound 29

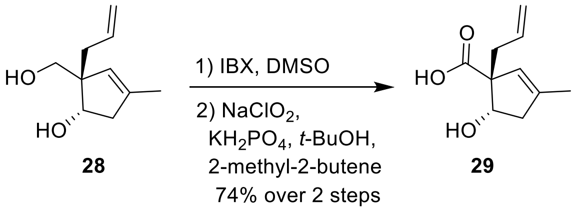

To a stirred solution of **28** (13 mg, 0.077 mmol) in DMSO (1 mL) at 19 °C was added IBX (43 mg, 0.15 mmol), the reaction mixture was stirred for 1 h and then quenched by saturated aqueous $NaHCO_3$ solution (5 mL). The aqueous layer was extracted with *t*-BuOMe (3 × 5 mL), then the combined organic layers were washed with brine (10 mL), dried over Na_2SO_4 and the solvent was removed to afford the crude aldehyde which was directly used in the next step.

*Use of the crude aldehyde is necessary due to its instability and it will partly decompose during flash column chromatography.

To a stirred solution of the above aldehyde intermediate in *t*-BuOH (1 mL) at 19 °C was added 2-methyl-2-butene (0.16 mL, 1.48 mmol), saturated aqueous KH_2PO_4 solution (0.2 mL) and $NaClO_2$ (34 mg, 0.37 mmol). The reaction mixture was stirred for 5 h and then the reaction mixture was diluted by EtOAc (3 mL) followed by addition of 1 mol/L HCl solution to adjust the pH of the organic phase below 3. After extracted with EtOAc (3 × 10 mL), the combined organic layers were washed with brine (10 mL) and dried over Na_2SO_4. The solvent was removed by rotary evaporation to give the crude product, which was purified through flash column chromatography (EtOAc/petroleum ether 1:1) to yield **29** (10 mg, 0.057 mmol, 74% over 2 steps) as a colorless oil. **29** was directly subjected to next step.

Synthesis of compound 13

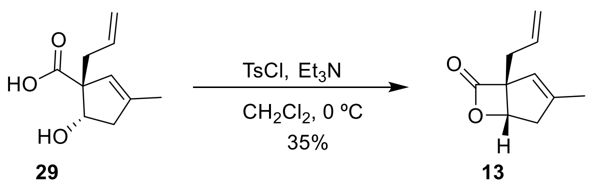

To a stirred solution of **29** (21 mg, 0.12 mmol) in dry CH_2Cl_2 (1.5 mL) at 0 °C was added Et_3N (48 μL, 0.33 mmol) and TsCl (24 mg, 0.13 mmol). After 1.5 h, the reaction mixture was diluted with CH_2Cl_2 (4 mL) and quenched with water (5 mL). The aqueous phase was extracted with *t*-BuOMe (3 × 10 mL). The combined organic phases were dried over anhydrous sodium sulfate, concentrated and purified through flash column chromatography (EtOAc/petroleum ether 1:40) to afford compound **13** (6.6 mg, 0.04 mmol, 35%) as a yellow oil.

Data for 13: R*f* = 0.78 (silica, EtOAc : petroleum ether = 1:4, stains with PMA). **^{1}H NMR (400MHz, Chloroform-*d*):** δ 5.83 – 5.71 (m, 1H), 5.34 – 5.30 (m, 1H), 5.19 – 5.11 (m, 2H), 4.80 – 4.76 (m, 1H), 2.66 (s, 2H), 2.64 – 2.58 (m, 1H), 2.53 – 2.46 (m, 1H), 1.82 – 1.79 (m, 3H) ppm. **^{13}C NMR (100MHz, Chloroform-*d*):** δ 173.3, 143.7, 132.3, 122.2, 119.0, 79.1, 74.8, 41.5, 33.7, 16.8 ppm. **HRMS(ESI)** for $C_{10}H_{13}O_2^+$ $[M+H]^+$: calcd. 165.0910, found 165.0915. **IR (neat) ν_{max}** 2927, 1820, 1639, 1401, 1113, 1018, 836 cm^{-1}.

3. ^{1}H and ^{13}C NMR spectra

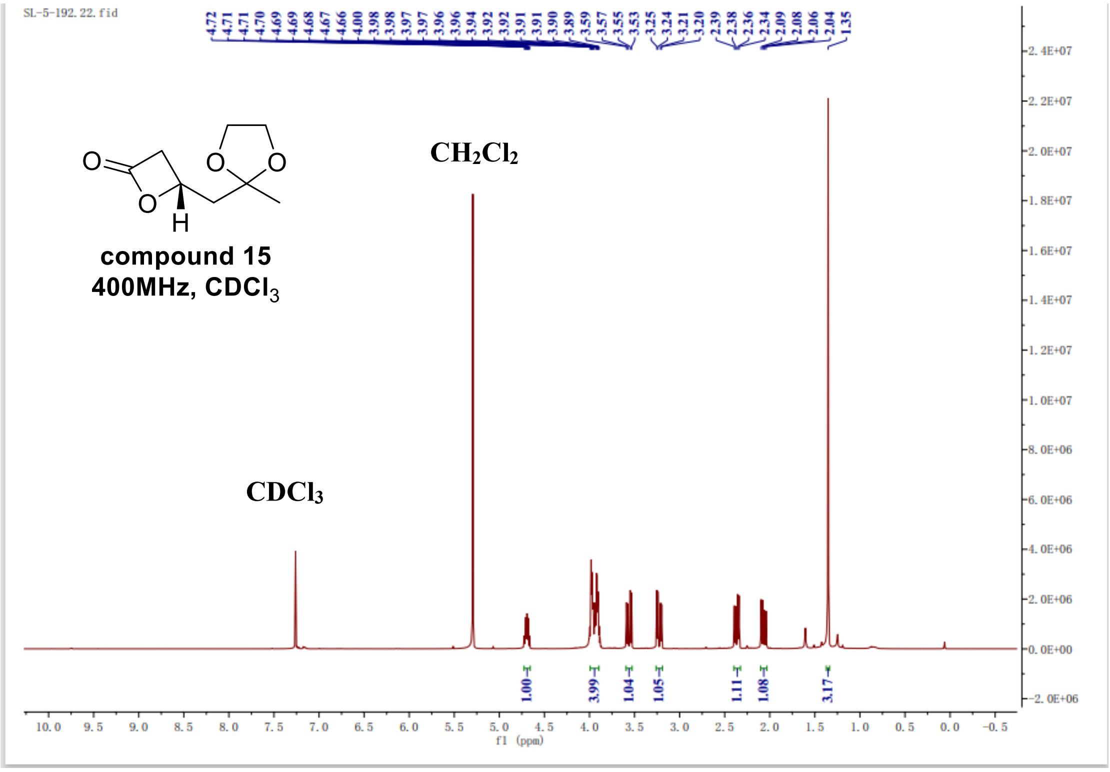

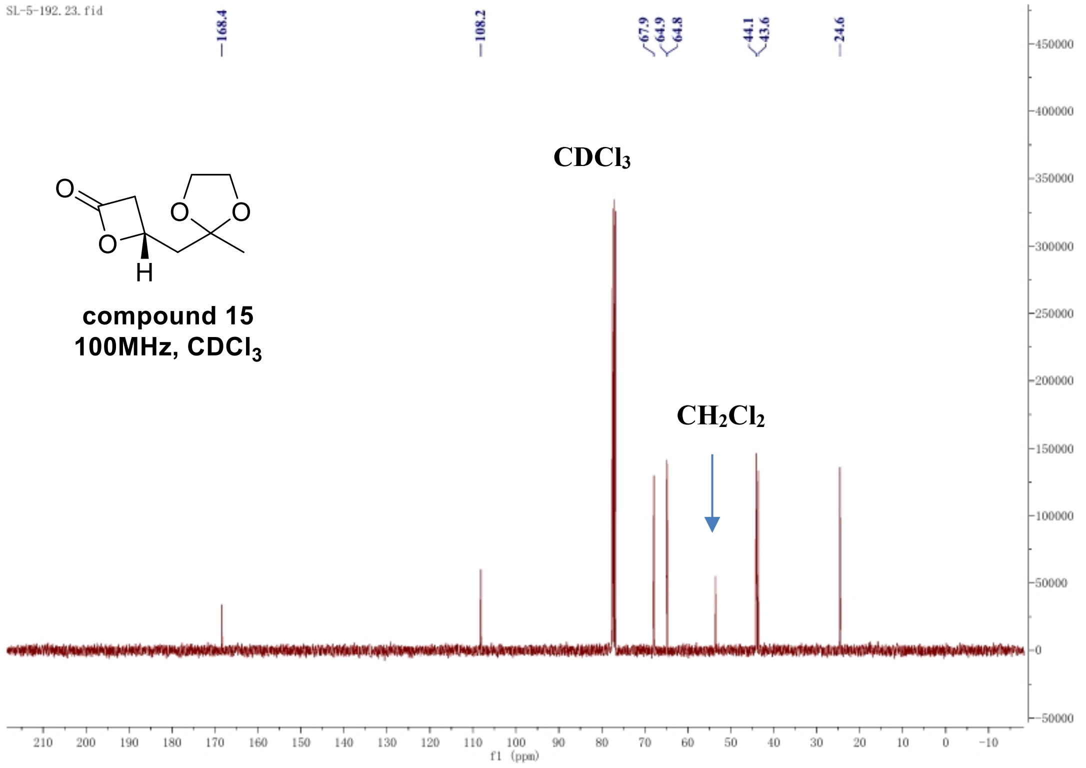

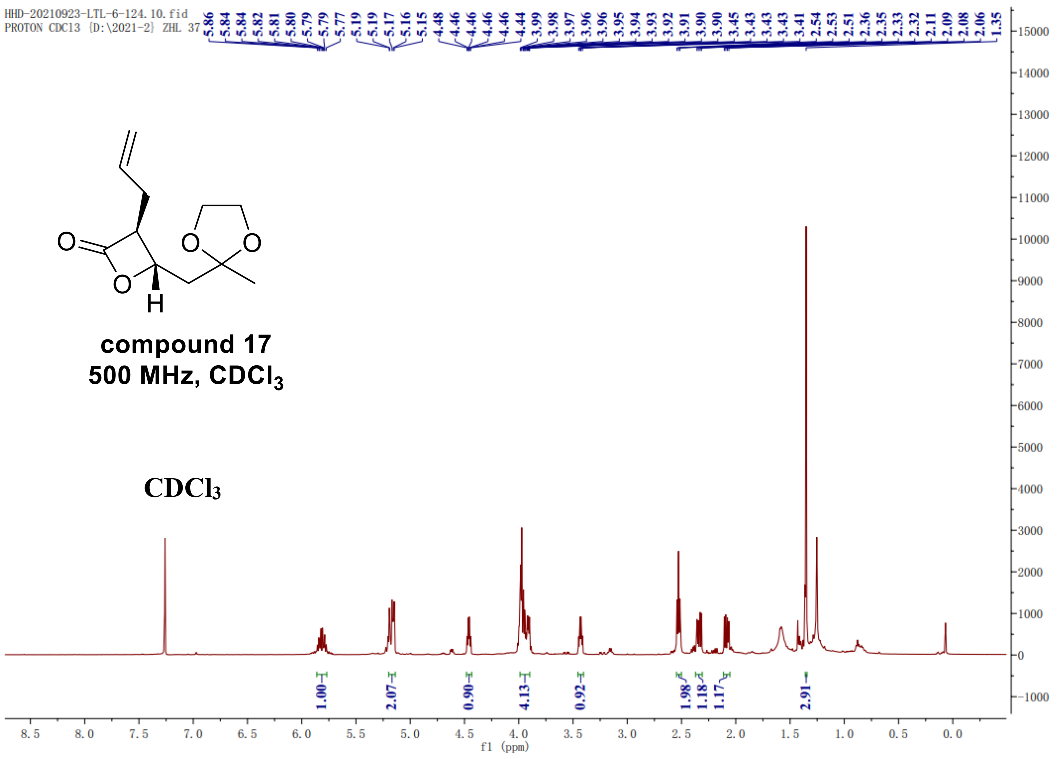

compound 17
500 MHz, CDCl3
CDCl3
f1 (ppm)

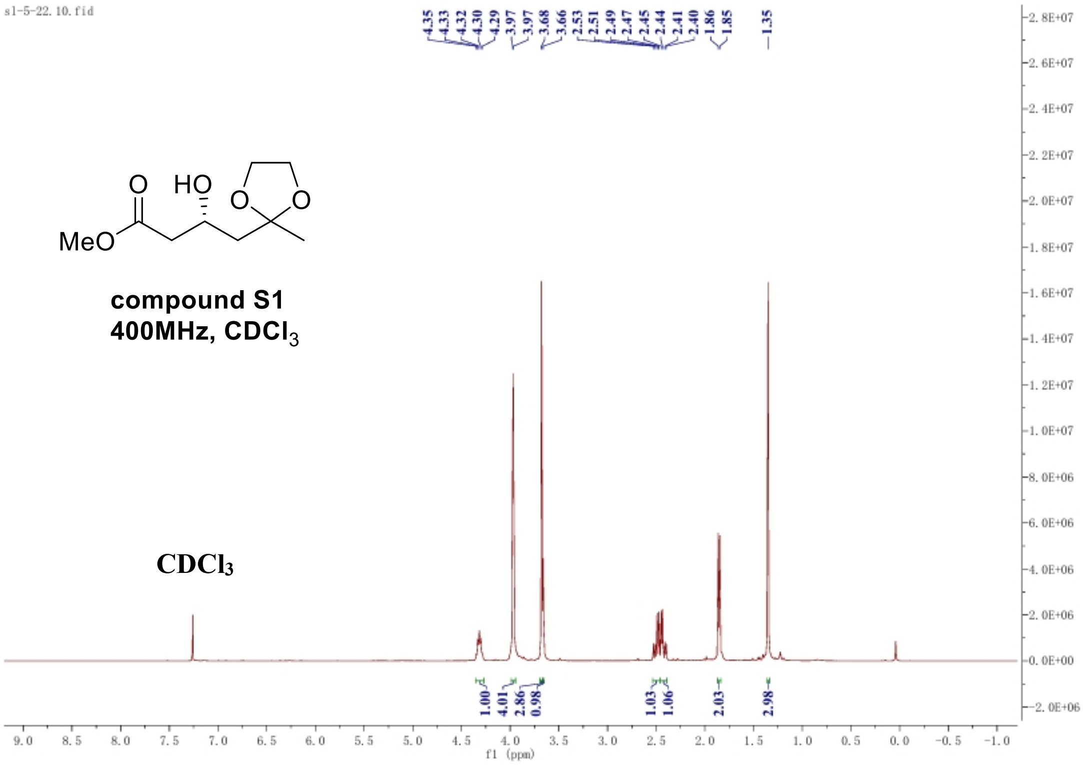
s1-5-22. 10. fid
4.35
4.33
4.32
4.30
4.29
3.97
3.97
3.68
3.66
2.53
2.51
2.49
2.47
2.45
2.44
2.41
2.40
1.86
1.85
1.35
MeO
O
HO
O
O
compound S1
400MHz, CDCl3
CDCl3
1.00
4.01
2.86
0.98
1.03
1.06
2.03
2.98
f1 (ppm)

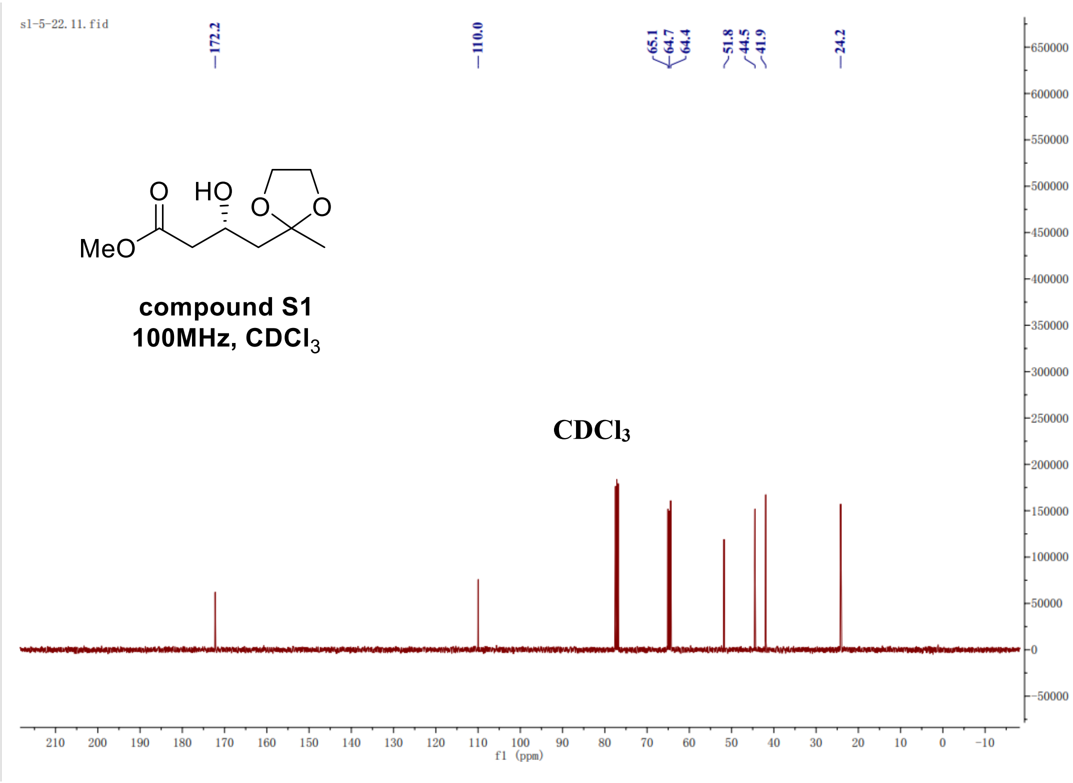
s1-5-22. 11. fid
172.2
110.0
65.1
64.7
64.4
51.8
44.5
41.9
24.2
MeO
O
HO
O
O
compound S1
100MHz, CDCl3
CDCl3
f1 (ppm)

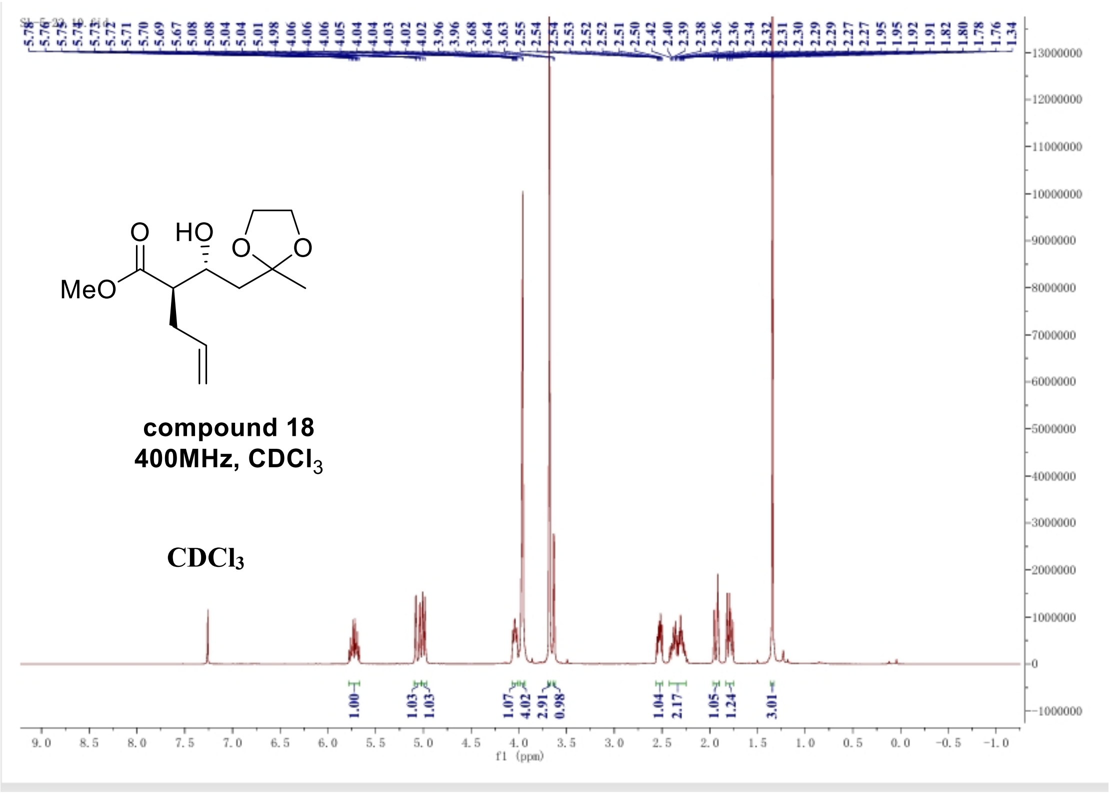
compound 18
400MHz, $CDCl_3$
$CDCl_3$
f1 (ppm)

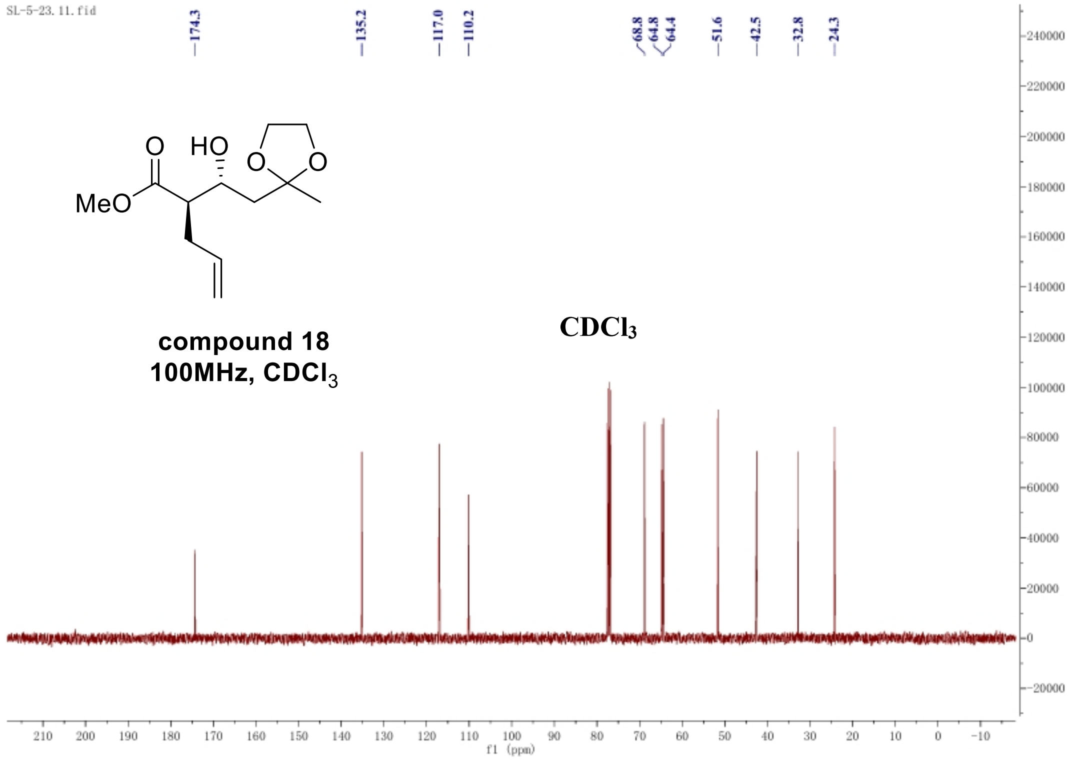
SL-5-23. 11. fid
174.3
135.2
117.0
110.2
68.8
64.8
64.4
51.6
42.5
32.8
24.3
compound 18
100MHz, $CDCl_3$
$CDCl_3$
f1 (ppm)

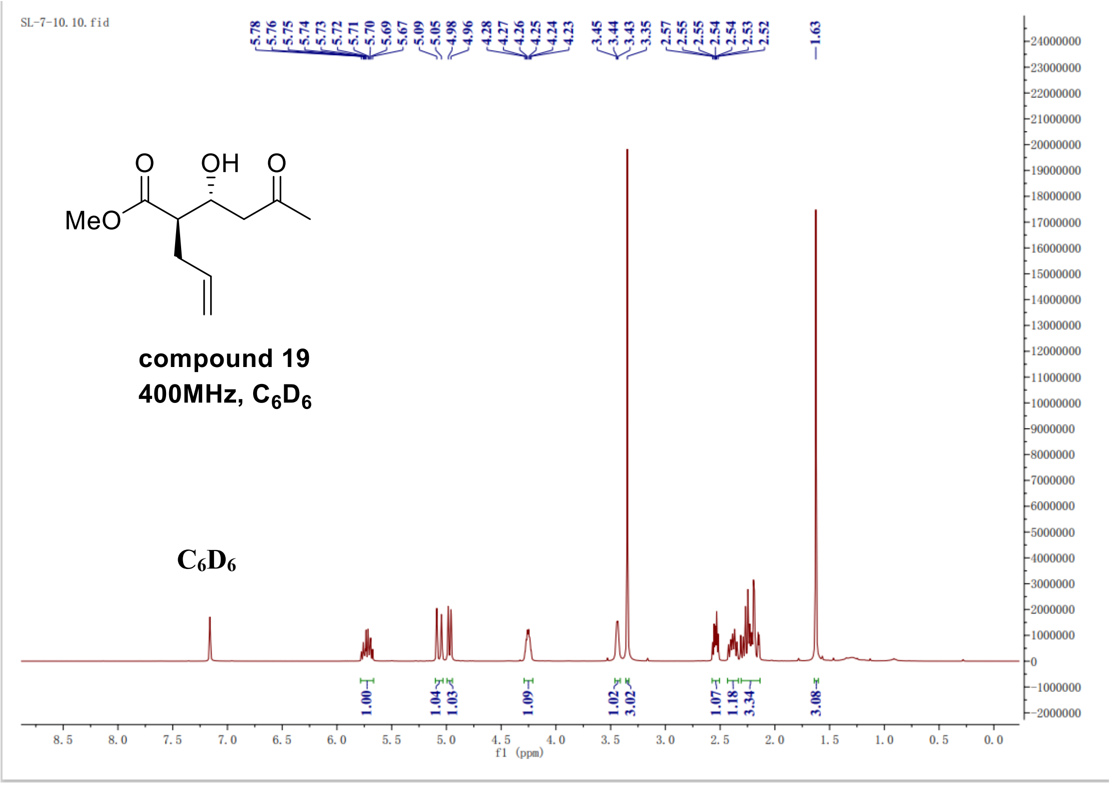
SL-7-10.10.fid
5.78
5.76
5.75
5.74
5.73
5.72
5.71
5.70
5.69
5.67
5.09
5.05
4.98
4.96
4.28
4.27
4.26
4.25
4.24
4.23
3.45
3.44
3.43
3.35
2.57
2.55
2.55
2.54
2.54
2.53
2.52
1.63
MeO
OH
compound 19
400MHz, C_6D_6
C_6D_6
1.00
1.04
1.03
1.09
1.02
3.02
1.07
1.18
3.34
3.08
f1 (ppm)

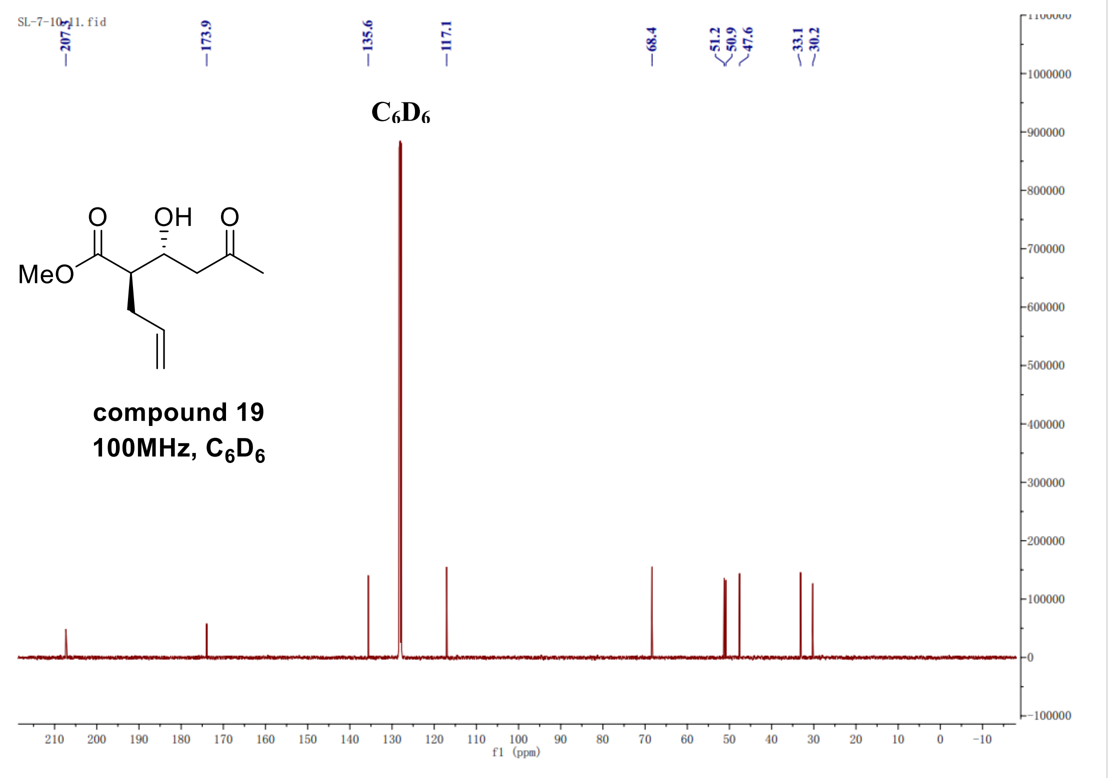
SL-7-10.11.fid
207.1
173.9
135.6
117.1
68.4
51.2
50.9
47.6
33.1
30.2
C_6D_6
MeO
OH
compound 19
100MHz, C_6D_6
f1 (ppm)

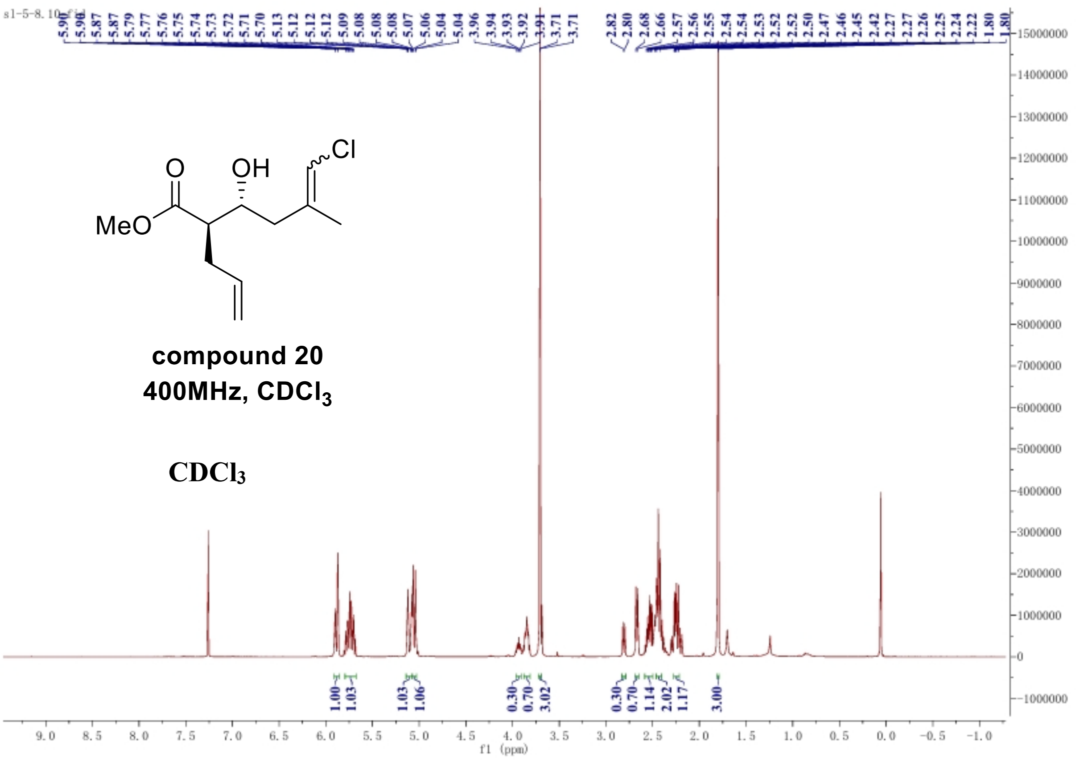
compound 20
400MHz, CDCl3
CDCl3
f1 (ppm)

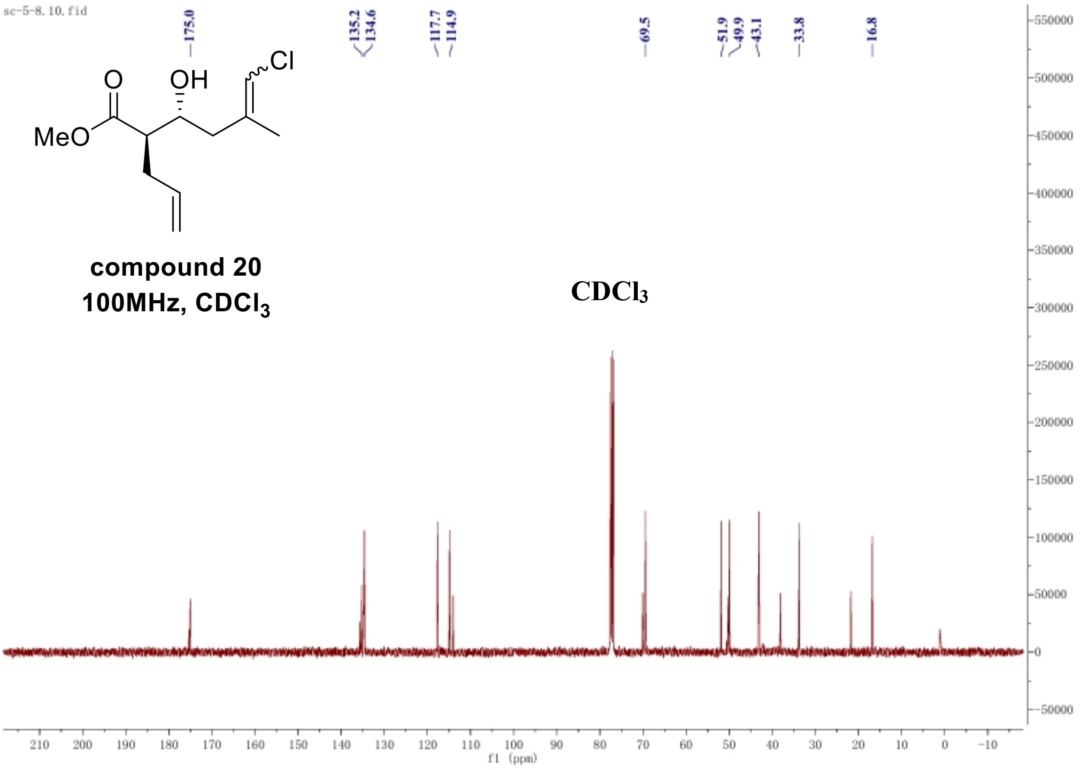
compound 20
100MHz, CDCl3
CDCl3
175.0
135.2
134.6
117.7
114.9
69.5
51.9
49.9
43.1
33.8
16.8
f1 (ppm)

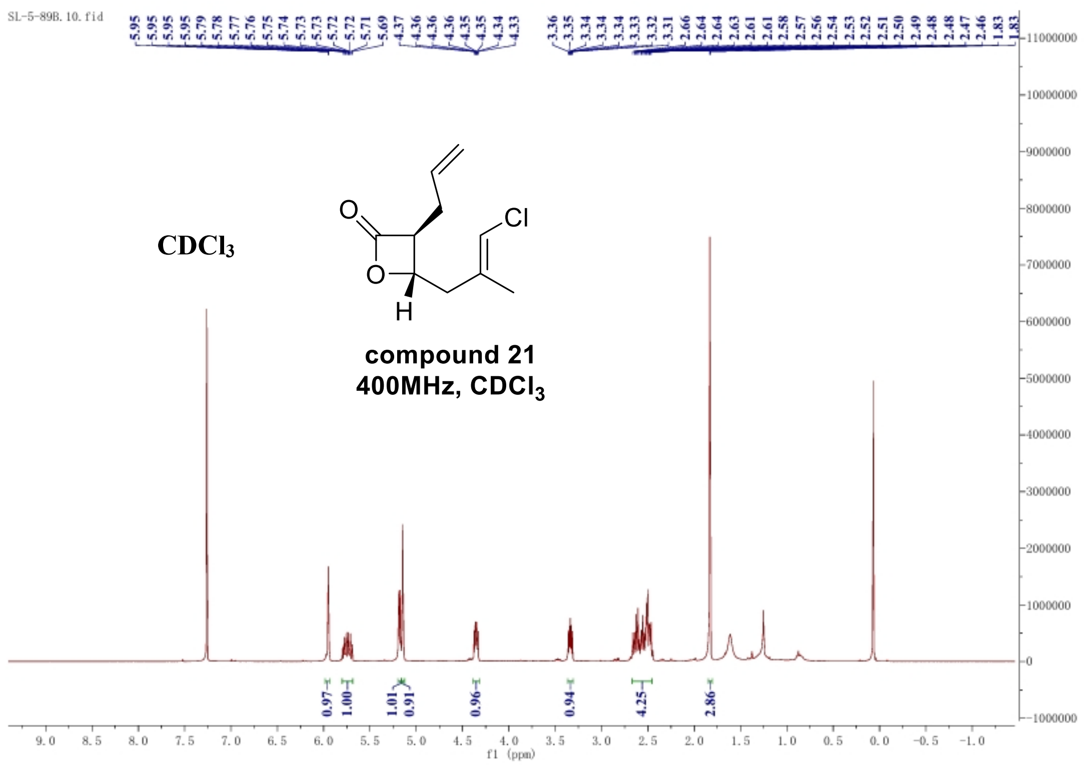
SL-5-89B.10.fid
CDCl3
Cl
O
O
H
compound 21
400MHz, CDCl3
0.97
1.00
1.01
0.91
0.96
0.94
4.25
2.86
f1 (ppm)

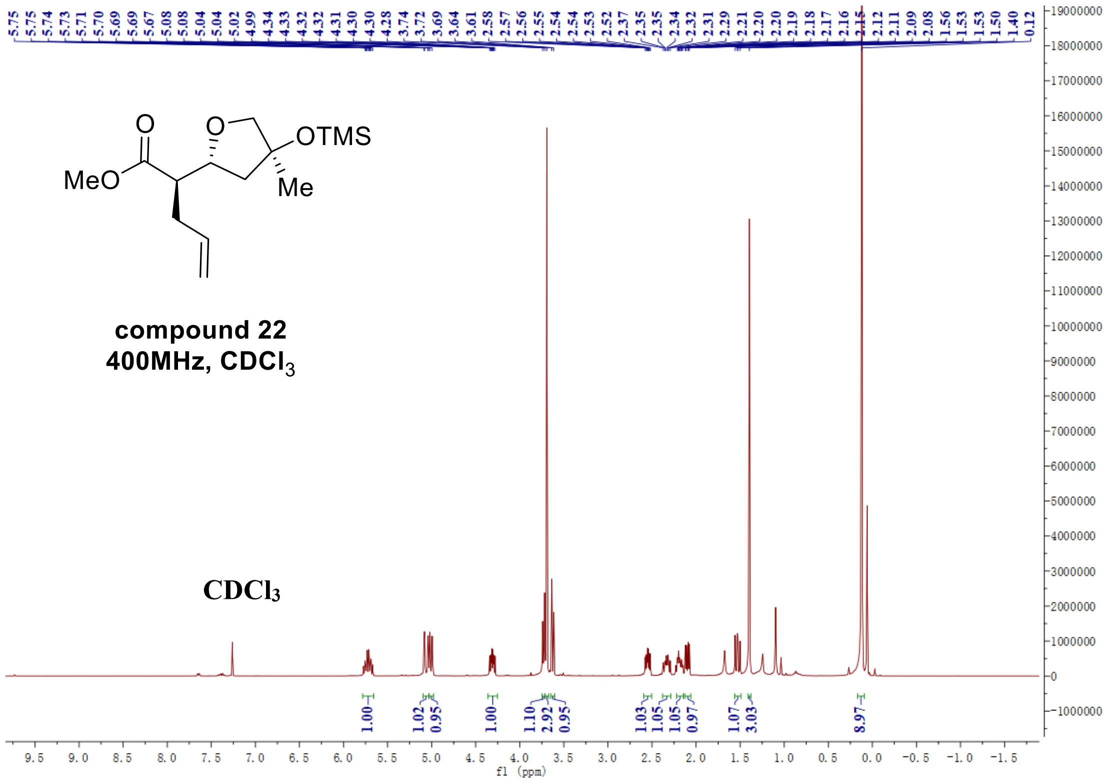
5.75 5.75 5.74 5.73 5.71 5.70 5.69 5.69 5.67 5.08 5.08 5.04 5.04 5.02 4.99 4.34 4.33 4.32 4.32 4.31 4.30 4.30 4.28 3.74 3.72 3.69 3.64 3.61 2.58 2.57 2.56 2.55 2.54 2.54 2.53 2.52 2.37 2.35 2.35 2.34 2.32 2.31 2.29 2.21 2.20 2.19 2.18 2.17 2.16 2.15 2.12 2.11 2.09 2.08 1.56 1.53 1.50 1.40 0.12
O
MeO
O
OTMS
Me
compound 22
400MHz, $CDCl_3$
$CDCl_3$
1.00 1.02 0.95 1.00 1.10 2.92 0.95 1.03 1.05 1.05 0.97 1.07 3.03 8.97
f1 (ppm)

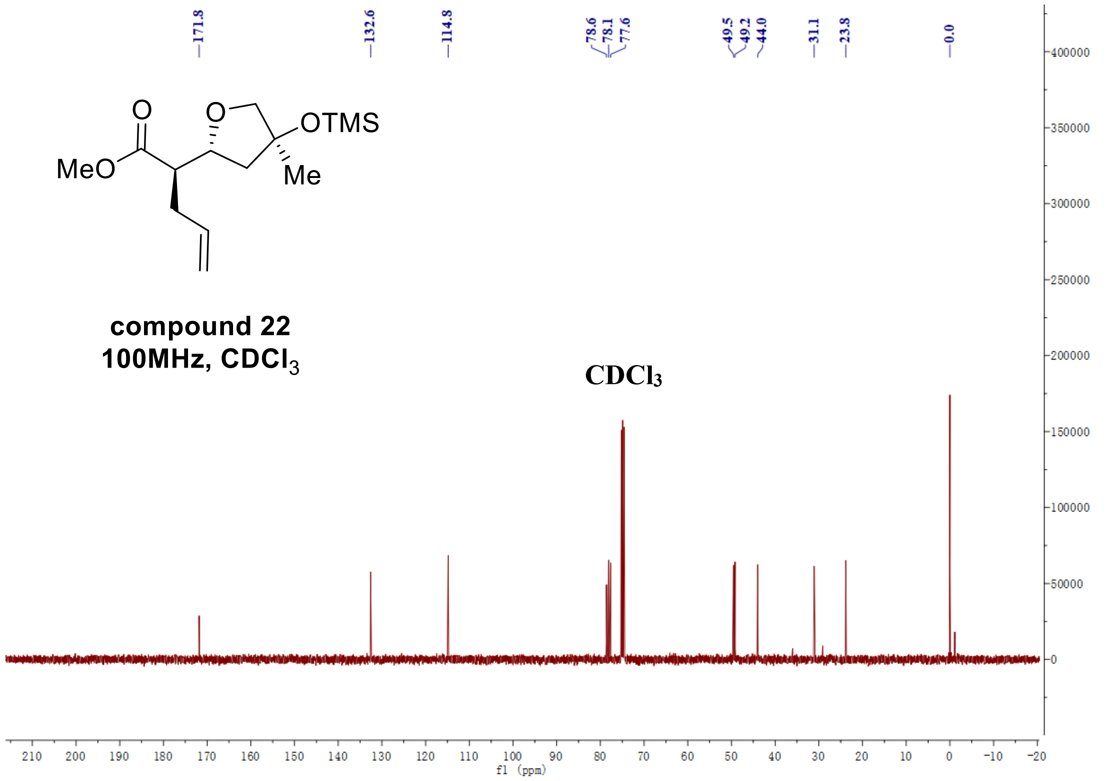
171.8 132.6 114.8 78.6 78.1 77.6 49.5 49.2 44.0 31.1 23.8 0.0
O
MeO
O
OTMS
Me
compound 22
100MHz, $CDCl_3$
$CDCl_3$
f1 (ppm)

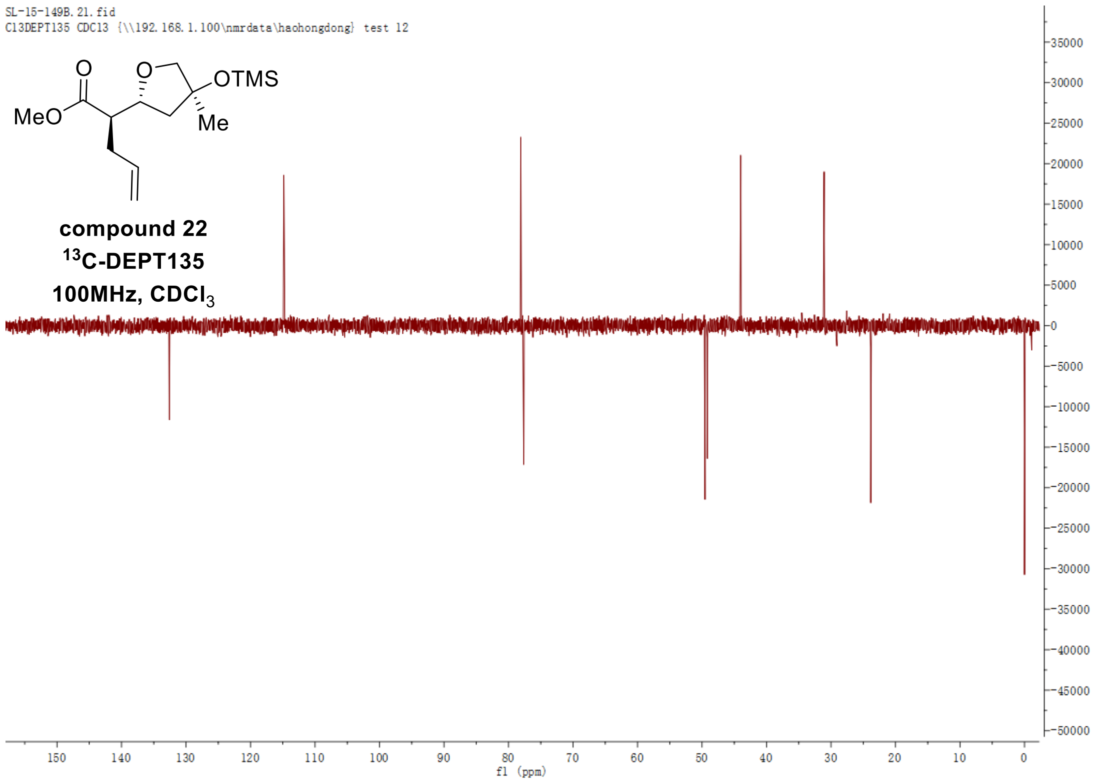
SL-15-149B.21.fid
C13DEPT135 CDCl3 {\\192.168.1.100\nmrdata\haohongdong} test 12
O
O
OTMS
MeO
Me
compound 22
13C-DEPT135
100MHz, CDCl3
f1 (ppm)

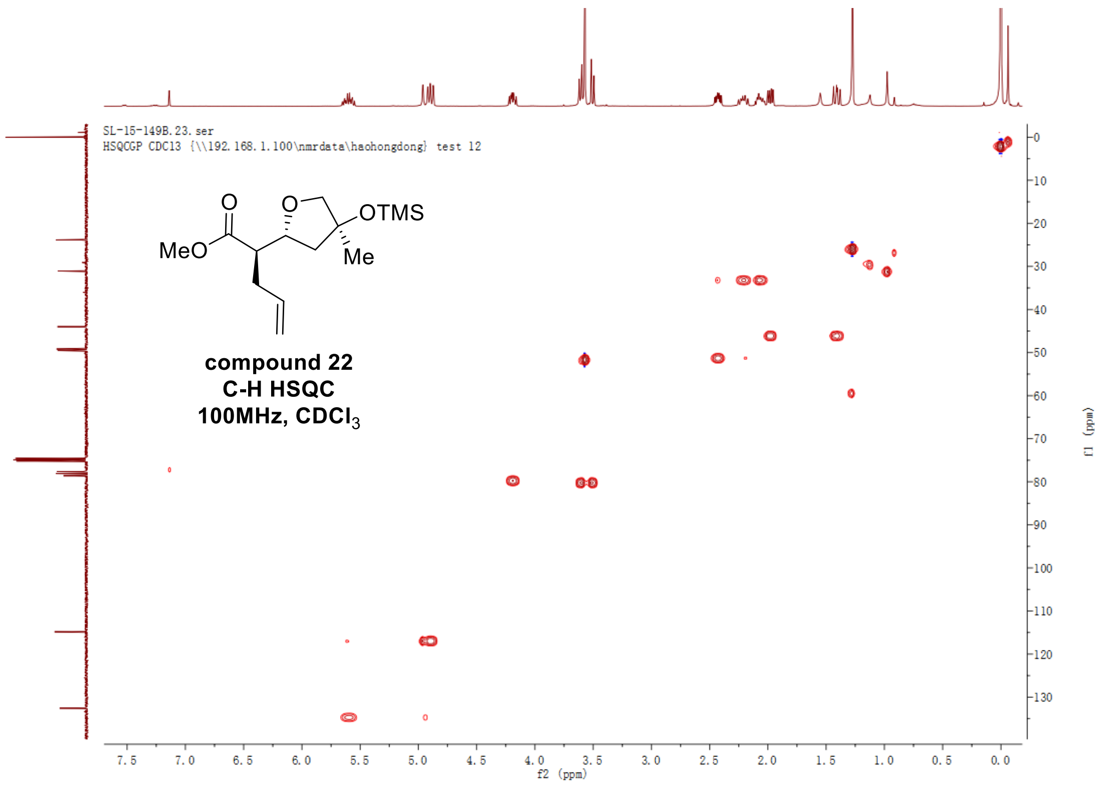
SL-15-149B.23.ser
HSQCGP CDCl3 {\\192.168.1.100\nmrdata\haohongdong} test 12
O
O
OTMS
MeO
Me
compound 22
C-H HSQC
100MHz, CDCl3
f2 (ppm)
f1 (ppm)

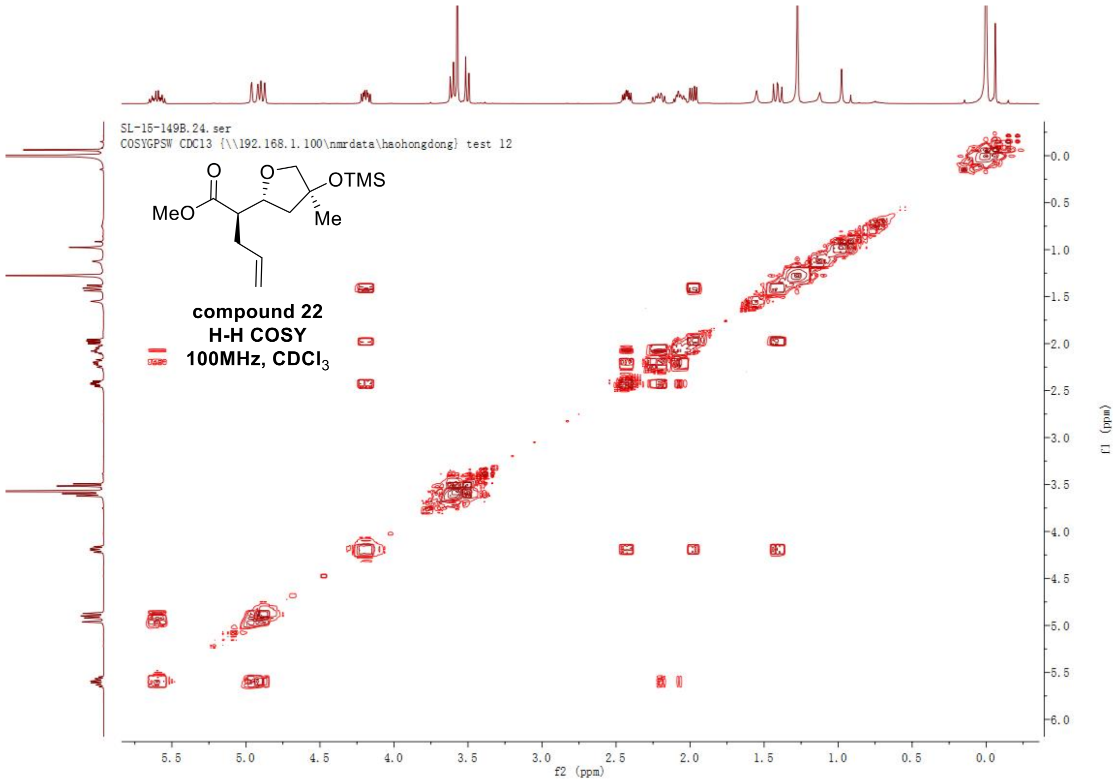
SL-15-149B.24.ser
COSYGPSW CDCl3 {\\192.168.1.100\nmrdata\haohongdong} test 12
OTMS
MeO
Me
compound 22
H-H COSY
100MHz, CDCl3
f2 (ppm)
f1 (ppm)

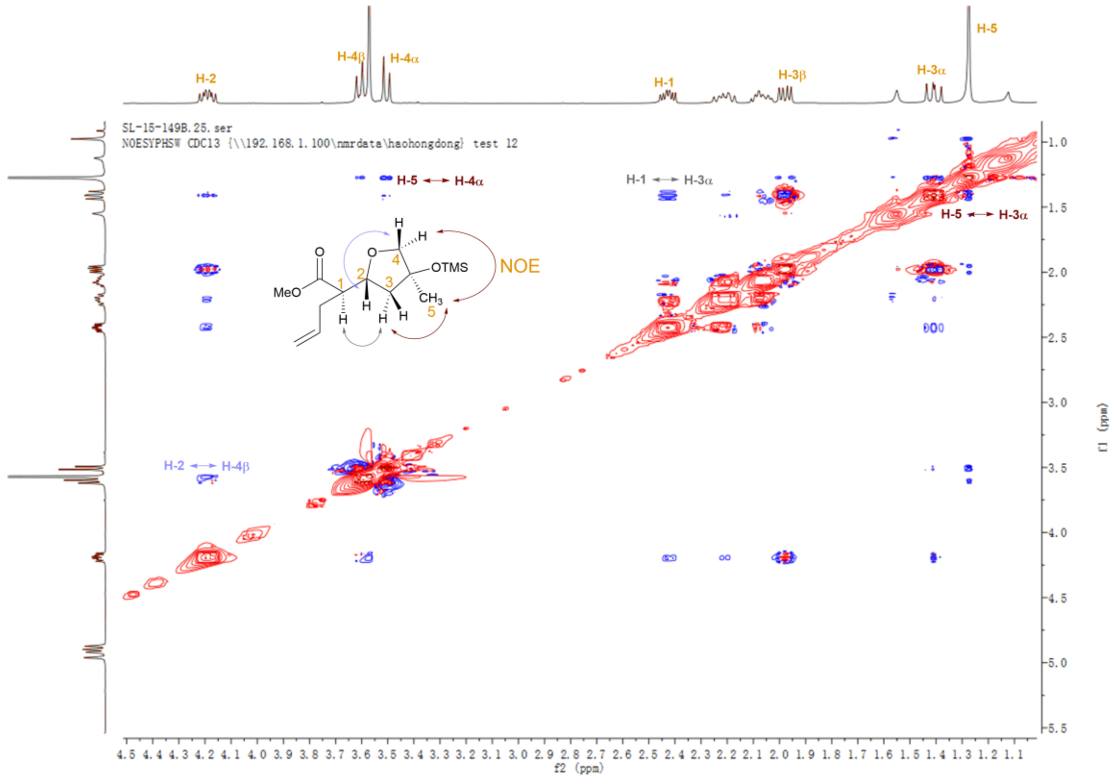
H-2
H-4β
H-4α
H-1
H-3β
H-3α
H-5
SL-15-149B.25.ser
NOESYPHSW CDCl3 {\\192.168.1.100\nmrdata\haohongdong} test 12
H-5 ⟷ H-4α
H-1 ⟷ H-3α
H-5 ⟷ H-3α
NOE
OTMS
MeO
CH3
H-2 ⟷ H-4β
f2 (ppm)
f1 (ppm)

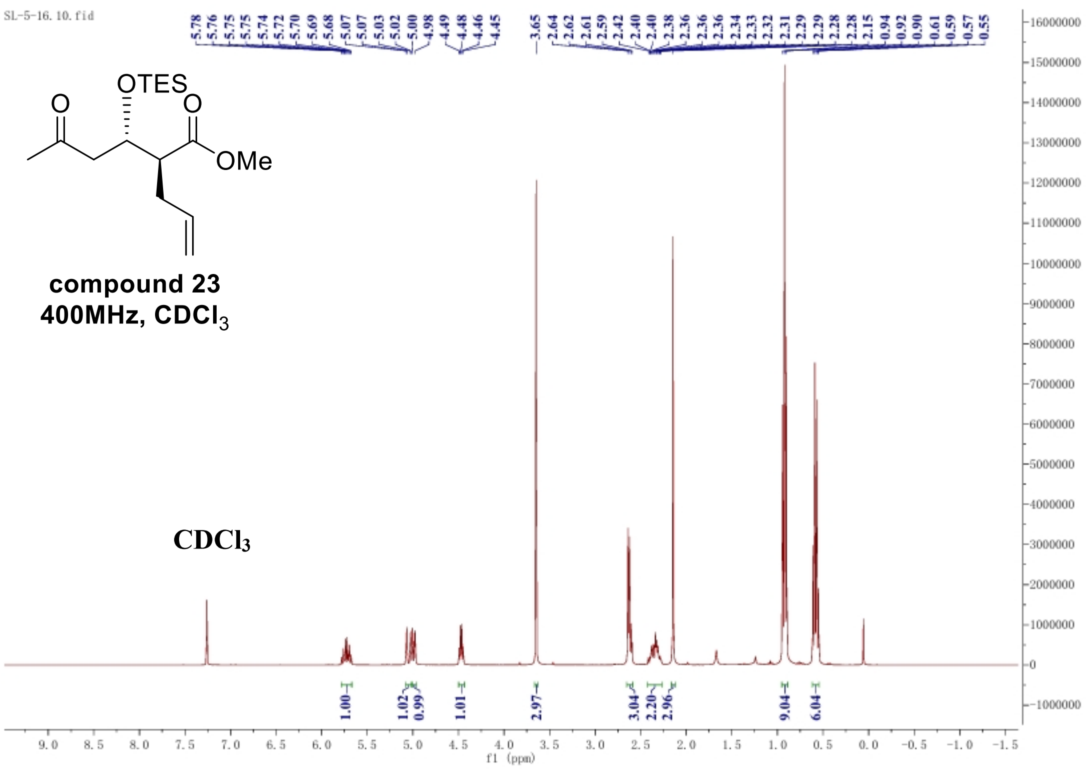

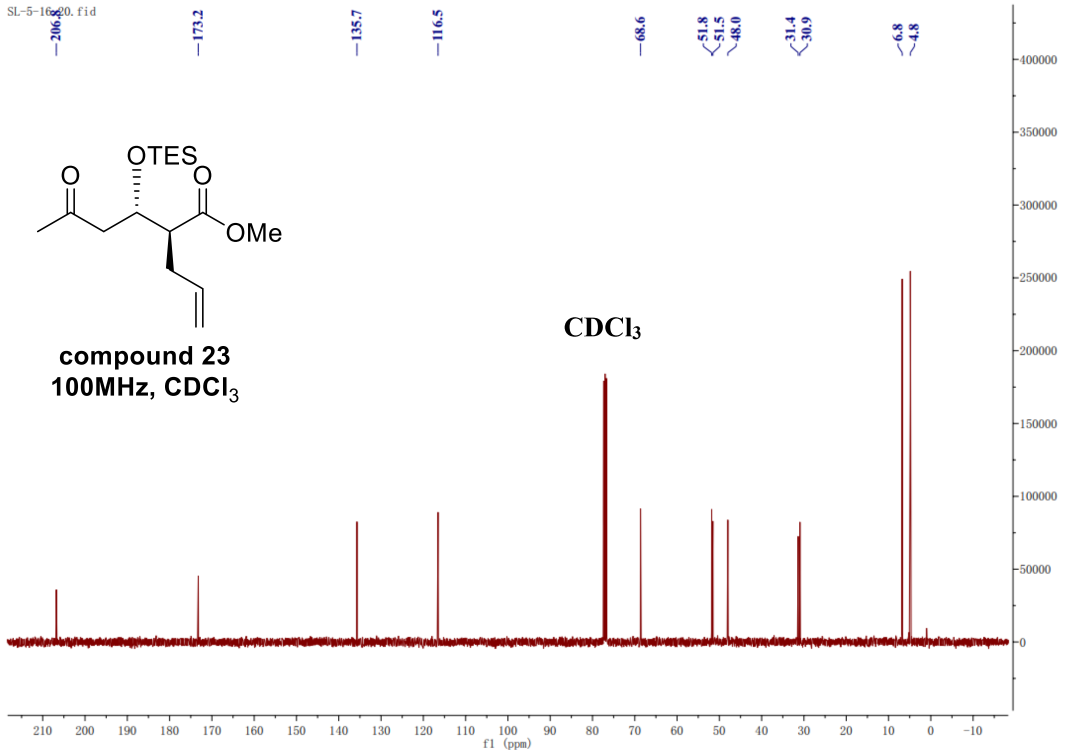

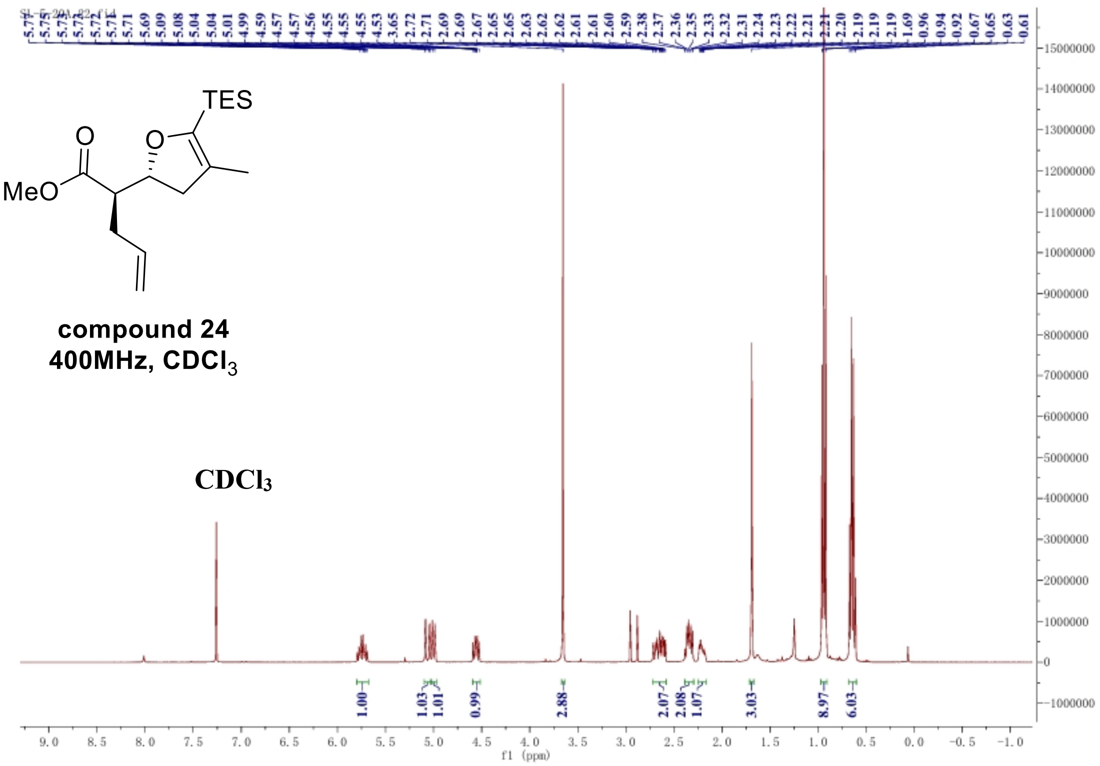
TES
O
MeO
O
compound 24
400MHz, $CDCl_3$
CDCl3
f1 (ppm)

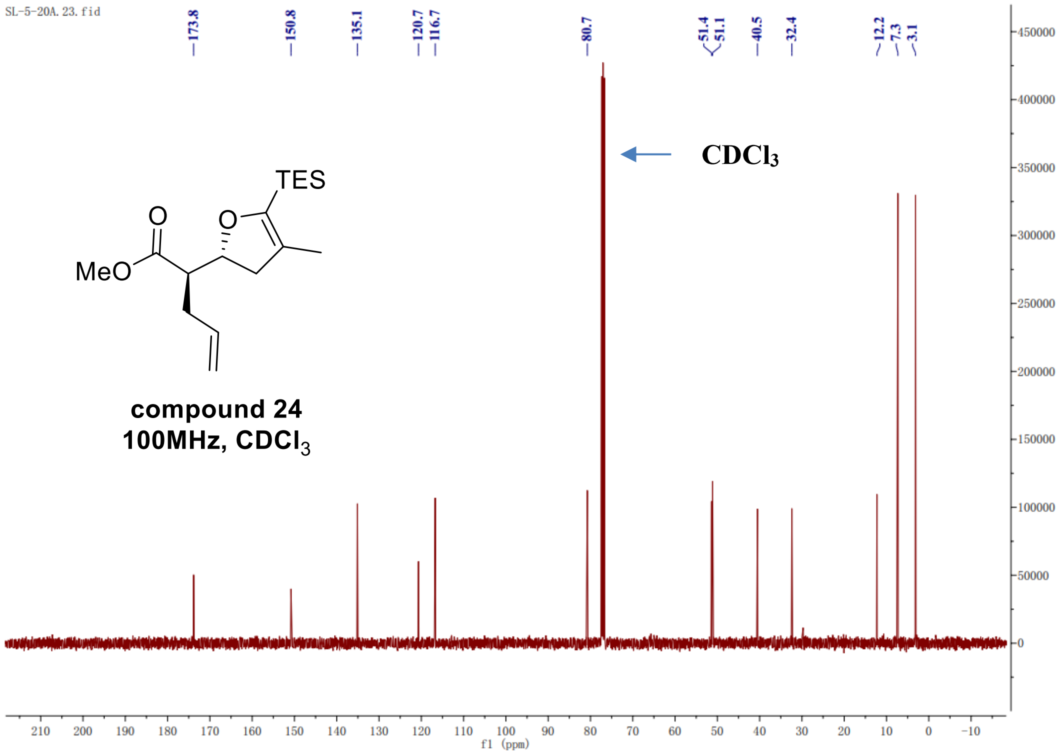
SL-5-20A.23.fid
173.8
150.8
135.1
120.7
116.7
80.7
51.4
51.1
40.5
32.4
12.2
7.3
3.1
CDCl3
TES
O
MeO
O
compound 24
100MHz, $CDCl_3$
f1 (ppm)

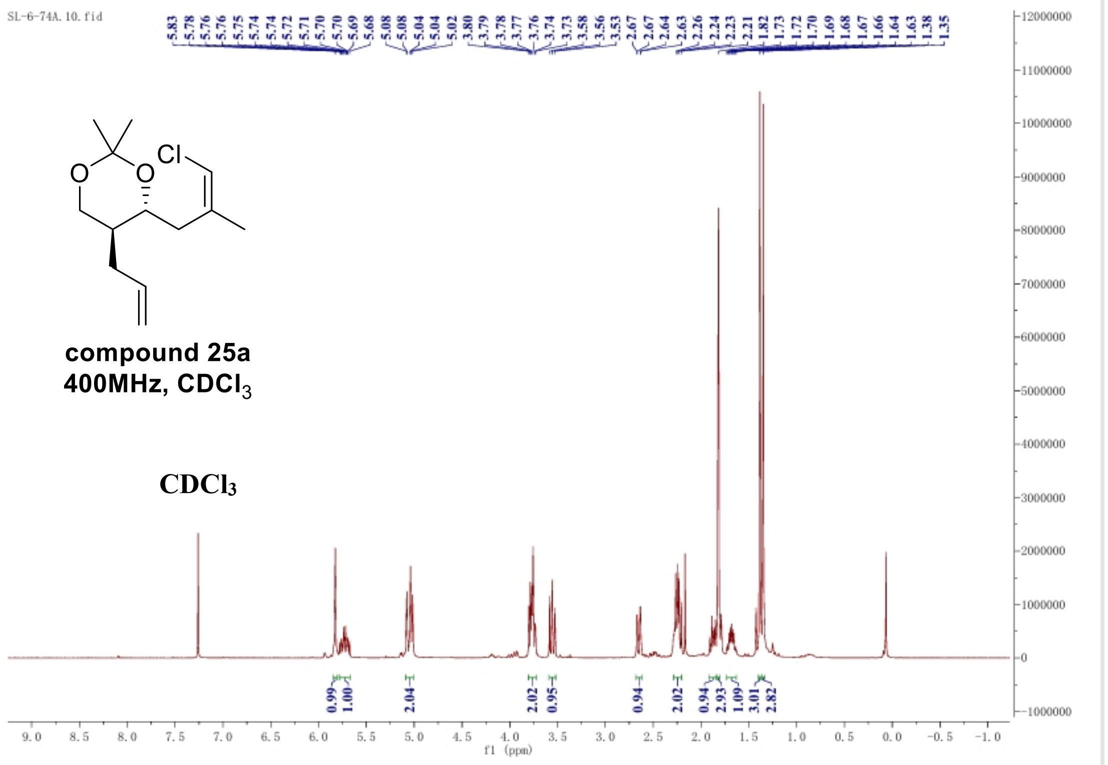
SL-6-74A. 10. fid
compound 25a
400MHz, CDCl3
CDCl3
0.99
1.00
2.04
2.02
0.95
0.94
2.02
0.94
2.93
1.09
3.01
2.82
f1 (ppm)

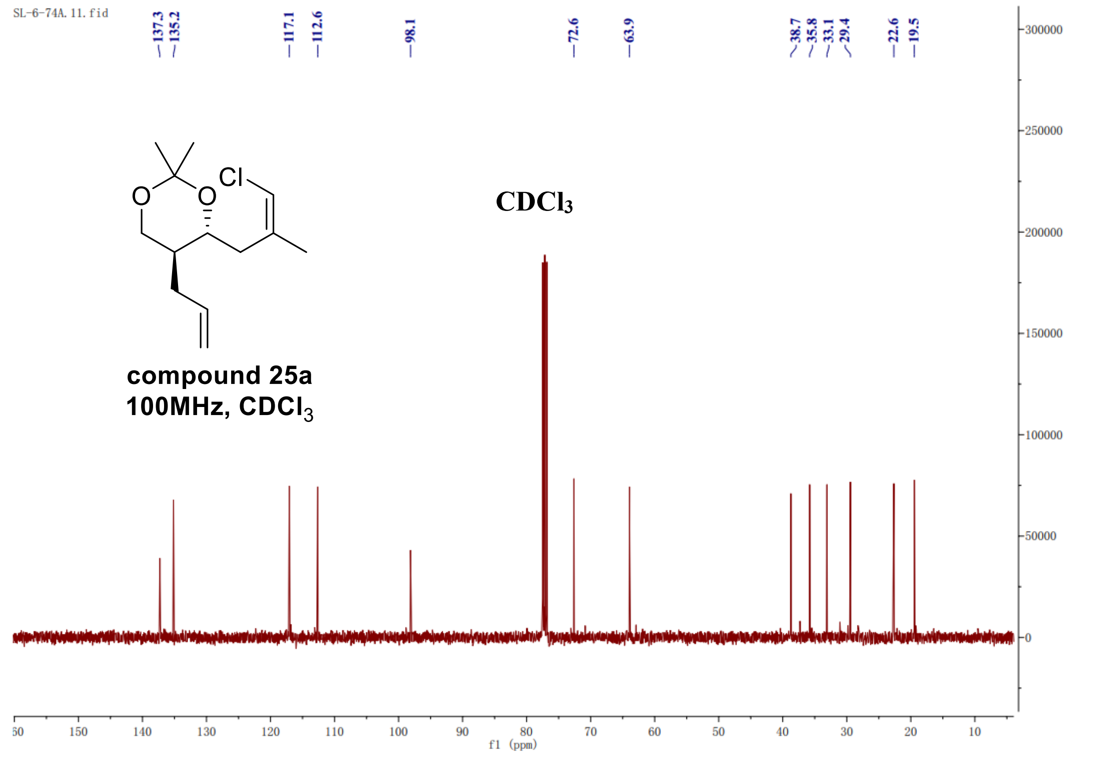
SL-6-74A. 11. fid
137.3
135.2
117.1
112.6
98.1
72.6
63.9
38.7
35.8
33.1
29.4
22.6
19.5
compound 25a
100MHz, CDCl3
CDCl3
f1 (ppm)

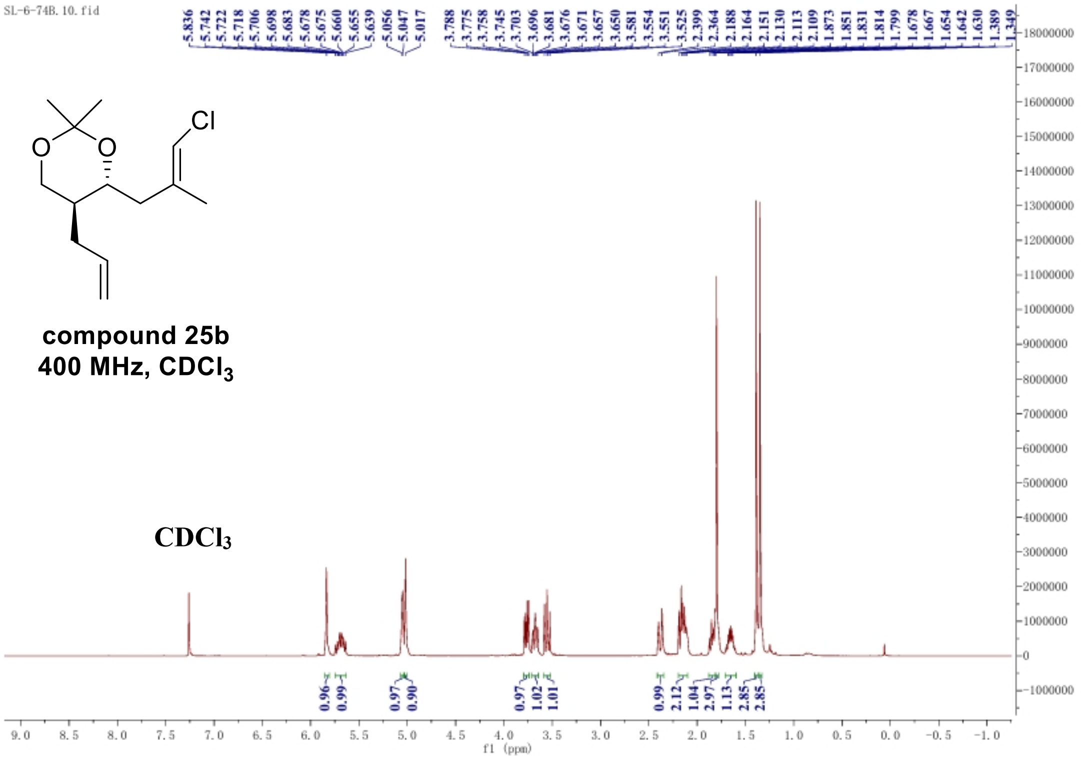

SL-6-74B.10.fid
compound 25b
400 MHz, $CDCl_3$
$CDCl_3$
f1 (ppm)

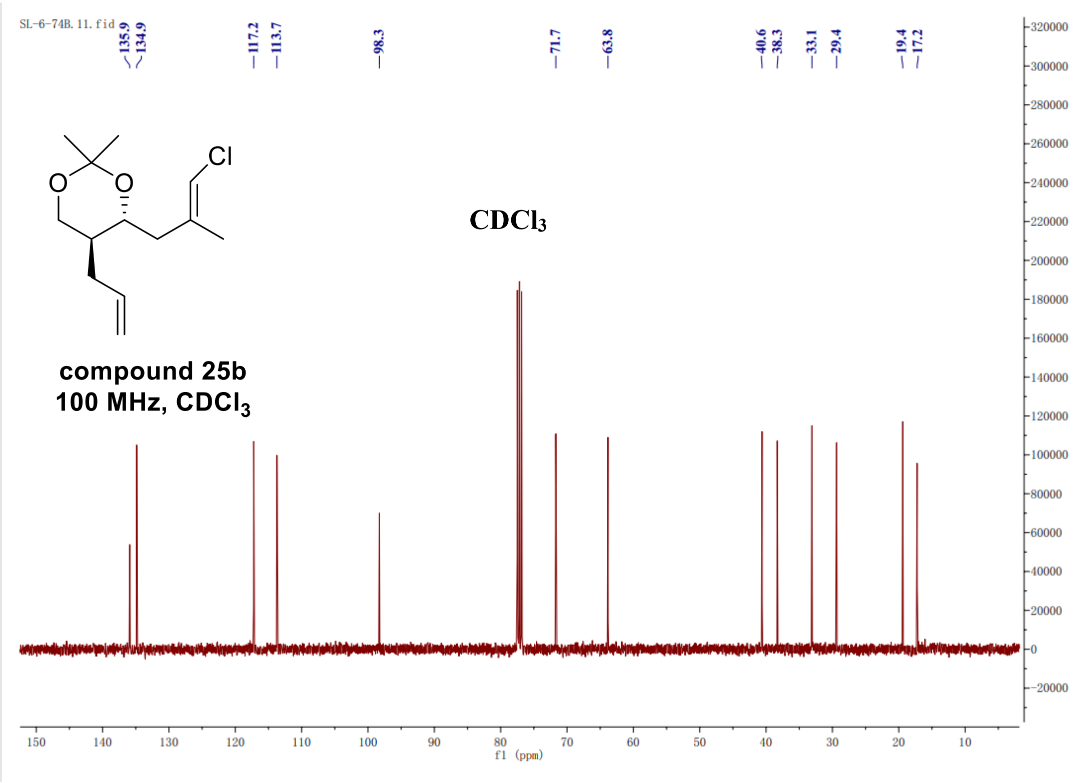

SL-6-74B.11.fid
135.9
134.9
117.2
113.7
98.3
71.7
63.8
40.6
38.3
33.1
29.4
19.4
17.2
compound 25b
100 MHz, $CDCl_3$
$CDCl_3$
f1 (ppm)

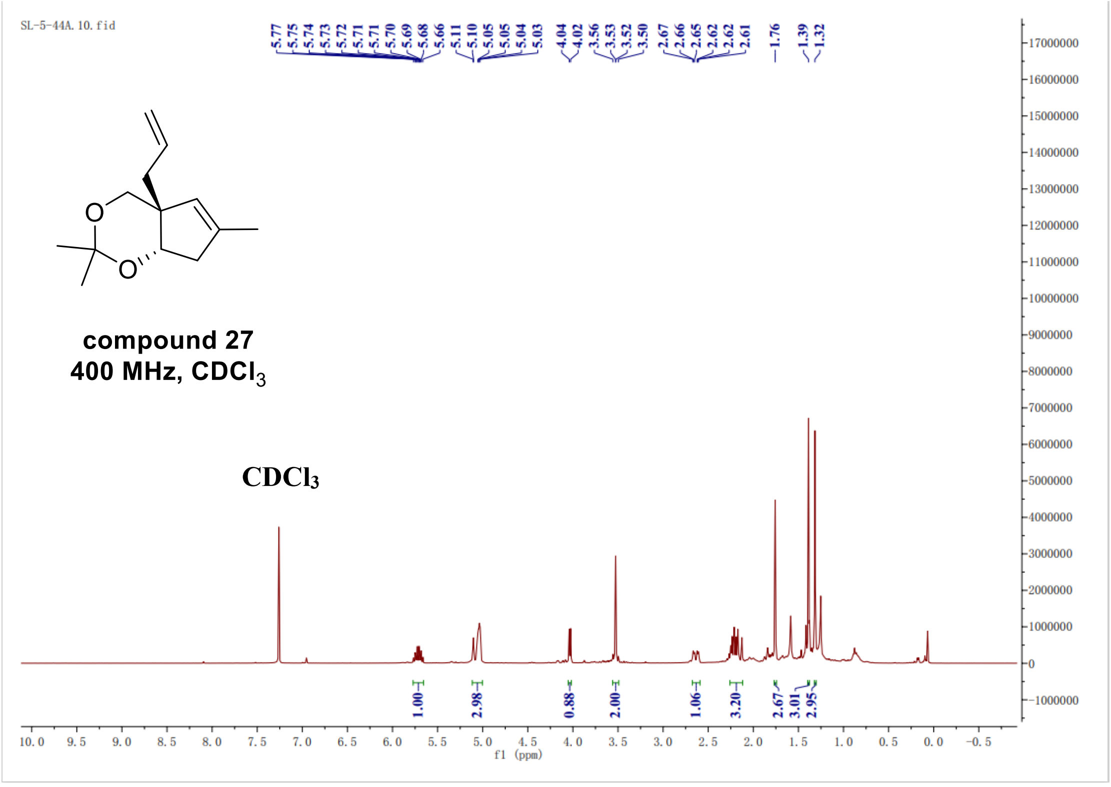
SL-5-44A.10.fid
compound 27
400 MHz, $CDCl_3$
$CDCl_3$
f1 (ppm)

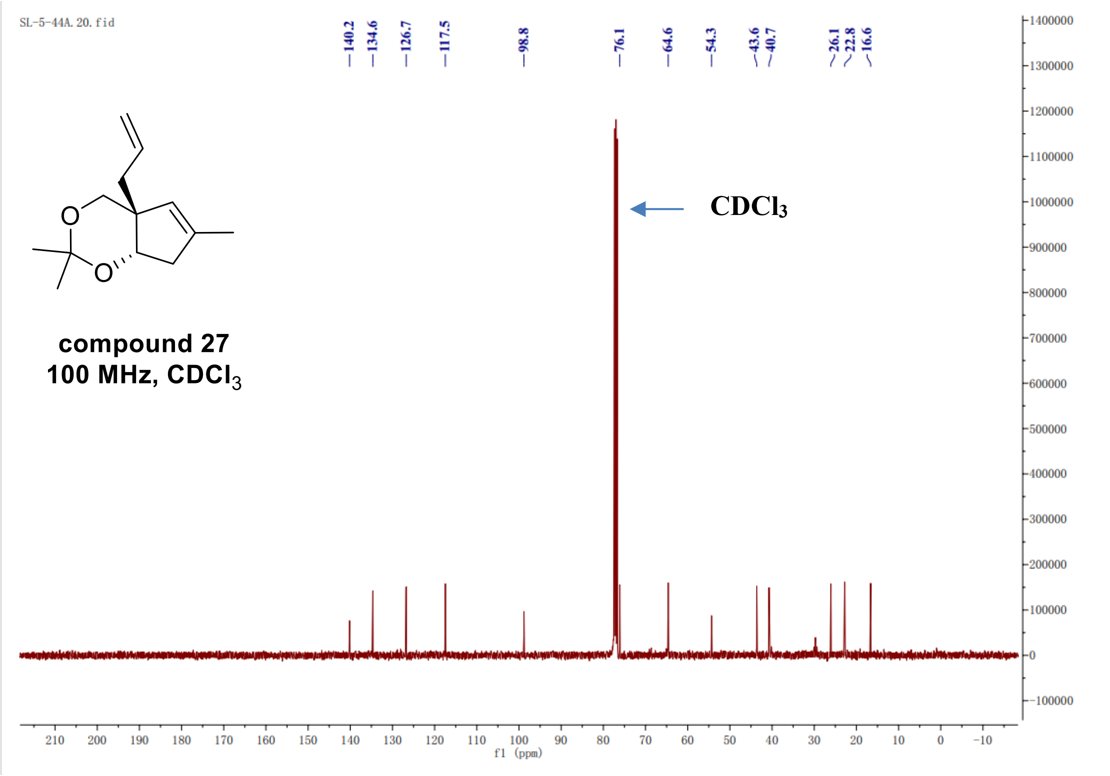
SL-5-44A.20.fid
140.2
134.6
126.7
117.5
98.8
76.1
64.6
54.3
43.6
40.7
26.1
22.8
16.6
compound 27
100 MHz, $CDCl_3$
$CDCl_3$
f1 (ppm)

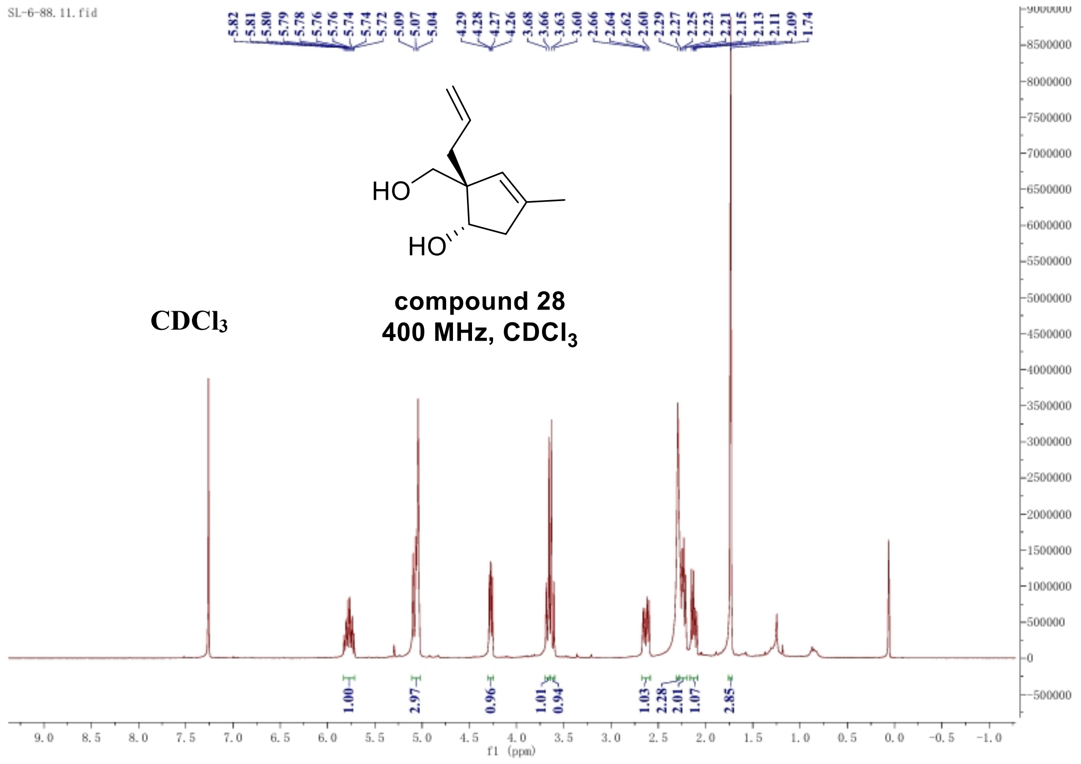
SL-6-88.11.fid
5.82
5.81
5.80
5.79
5.78
5.76
5.76
5.74
5.74
5.72
5.09
5.07
5.04
4.29
4.28
4.27
4.26
3.68
3.66
3.63
3.60
2.66
2.64
2.62
2.60
2.29
2.27
2.25
2.23
2.21
2.15
2.13
2.11
2.09
1.74
HO
HO
compound 28
400 MHz, CDCl3
CDCl3
1.00
2.97
0.96
1.01
0.94
1.03
2.28
2.01
1.07
2.85
f1 (ppm)

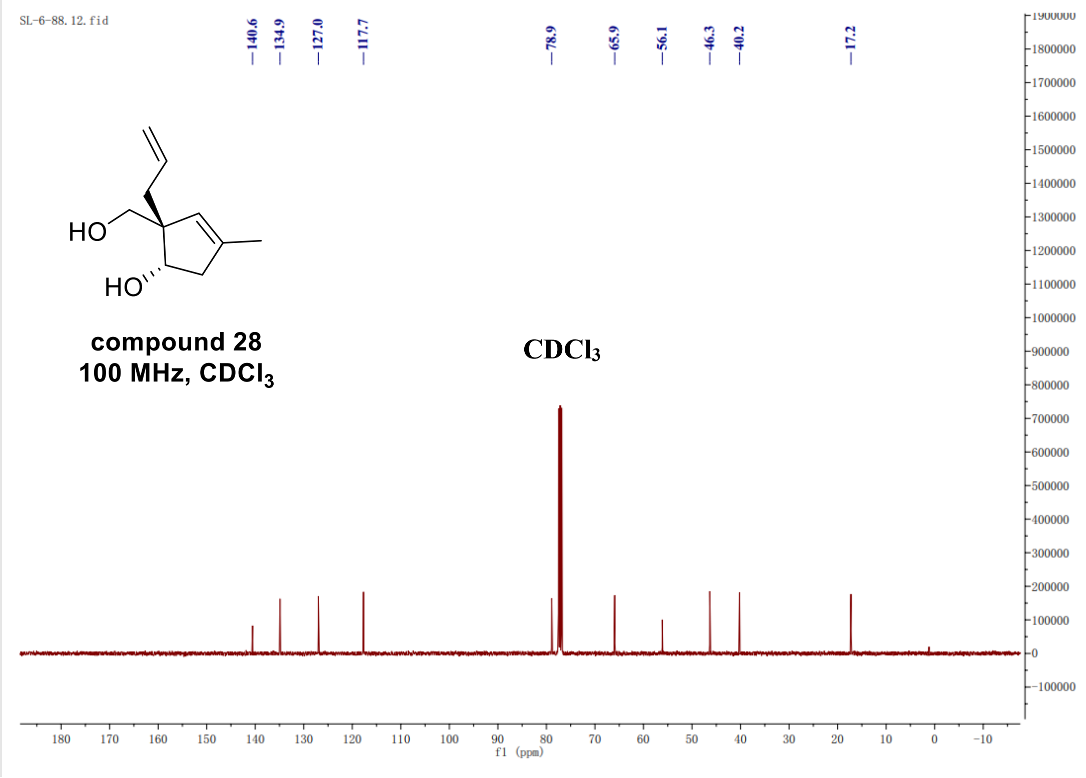
SL-6-88.12.fid
140.6
134.9
127.0
117.7
78.9
65.9
56.1
46.3
40.2
17.2
HO
HO
compound 28
100 MHz, CDCl3
CDCl3
f1 (ppm)

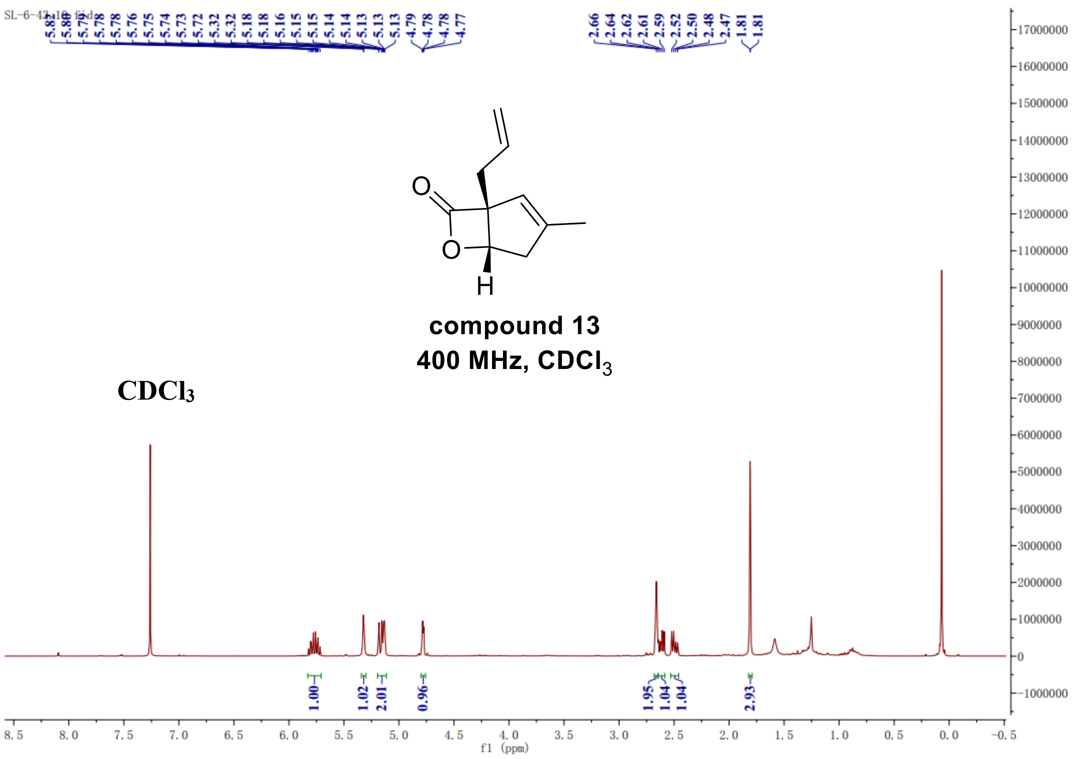
SL-6-43.10.fid
compound 13
400 MHz, CDCl3
CDCl3
f1 (ppm)
1.00
1.02
2.01
0.96
1.95
1.04
1.04
2.93

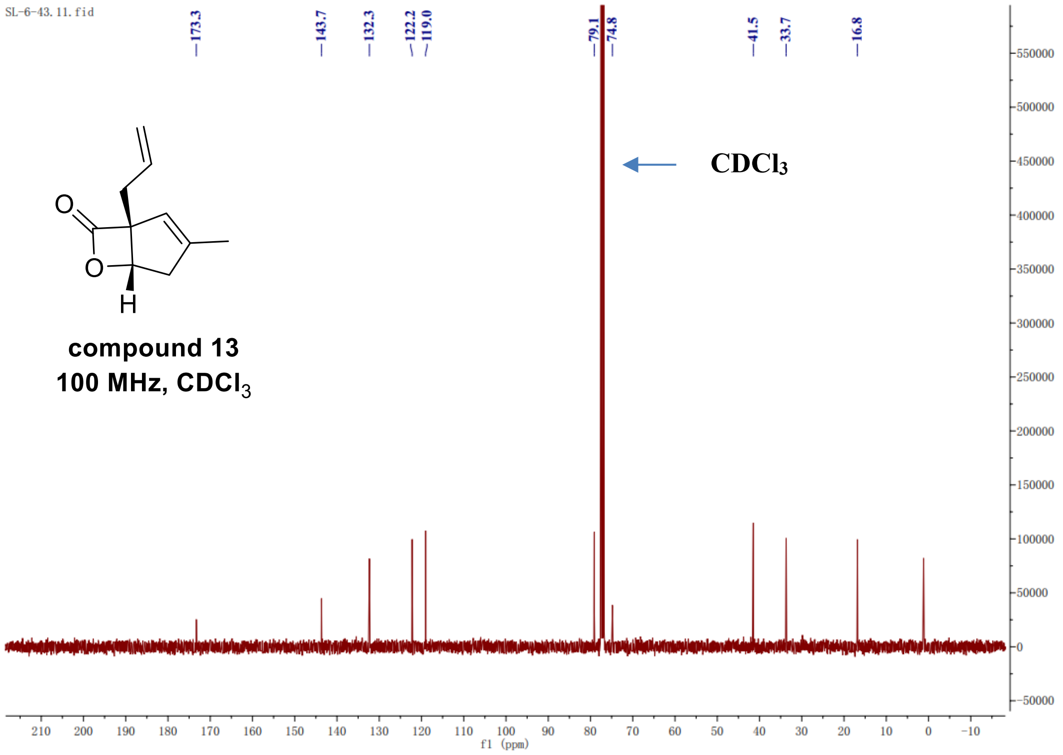
SL-6-43.11.fid
173.3
143.7
132.3
122.2
119.0
79.1
74.8
41.5
33.7
16.8
CDCl3
compound 13
100 MHz, CDCl3
f1 (ppm)
